# The Effect of Exposure to Neighborhood Violence on Glucocorticoid Receptor Signaling in Lung Tumors

**DOI:** 10.1158/2767-9764.CRC-24-0032

**Published:** 2024-07-03

**Authors:** Hannah Heath, Jin Y. Yoo, Sabrina Akter, Atharva Jain, Vani Sharma, Hannah McGee, Aiman Soliman, Abeer M. Mahmoud, Alicia K. Matthews, Robert A. Winn, Zeynep Madak-Erdogan, Sage J. Kim

**Affiliations:** 1 Department of Food Science and Human Nutrition, University of Illinois Urbana-Champaign, Urbana, Illinois.; 2 School of Molecular and Cellular Biology, College of Liberal Arts and Sciences, University of Illinois Urbana-Champaign, Urbana, Illinois.; 3 National Center for Supercomputing Applications, University of Illinois Urbana-Champaign, Urbana, Illinois.; 4 Department of Medicine, University of Illinois Chicago, Chicago, Illinois.; 5 Department of Kinesiology and Nutrition, University of Illinois Chicago, Chicago, Illinois.; 6 School of Nursing, Columbia University, New York, New York.; 7 Massey Cancer Center, Virginia Commonwealth University, Richmond, Virginia.; 8 Cancer Center at Illinois, University of Illinois Urbana-Champaign, Urbana, Illinois.; 9 Institute for Genomic Biology, University of Illinois Urbana-Champaign, Urbana, Illinois.; 10 School of Public Health, University of Illinois Chicago, Chicago, Illinois.; 11 University of Illinois Cancer Center, Chicago, Illinois.

## Abstract

Despite lower rates and intensity of smoking, Black men experience a higher incidence of lung cancer compared to white men. The racial disparity in lung cancer is particularly pronounced in Chicago, a highly segregated urban city. Neighborhood conditions, particularly social stress, may play a role in lung tumorigenesis. Preliminary studies indicate that Black men residing in neighborhoods with higher rates of violent crime have significantly higher levels of hair cortisol, an indicator of stress response. To examine the relationship between social stress exposure and gene expression in lung tumors, we investigated glucocorticoid receptor (GR) binding in 15 lung tumor samples in relation to GR target gene expression levels and zip code level residential violent crime rates. Spatial transcriptomics and a version of ChIP sequencing known as CUT&RUN were used. Heatmap of genes, pathway analysis, and motif analysis were conducted at the statistical significance of *P* < 0.05. GR recruitment to chromatin was correlated with zip code level residential violent crime rate and overall GR binding increased with higher violent crime rates. Our findings suggest that exposure to residential violent crime may influence tumor biology via reprogramming GR recruitment. Prioritizing lung cancer screening in neighborhoods with increased social stress, such as high levels of violent crime, may reduce racial disparities in lung cancer.

**Significance:**

Exposure to neighborhood violent crime is correlated with glucocorticoid signaling and lung tumor gene expression changes associated with increased tumor aggressiveness, suggesting social conditions have downstream biophysical consequences that contribute to lung cancer disparities.

## Introduction

Lung cancer is the leading cause of cancer-related deaths worldwide and is responsible for nearly 25% of all cancer deaths in the US. (1). Despite significant advancements in diagnosis and treatment, racial disparities in lung cancer persist. Particularly, Black men experience higher incidence and mortality rates of lung cancer compared to White men, while Black patients with lung cancer who smoke less and begin smoking later in their lives compared to White patients (2–4). Consequently, Black smokers are less likely to meet the screening eligibility criteria that focus on smoking history and age. A cohort study including 48,364 adult smokers found that, among those diagnosed with lung cancer, only 32% of Black smokers were eligible for screening compared to 56% of White smokers (5). While the frequency and intensity of smoking are lower for Blacks than Whites, the lung cancer rate is higher for Blacks, which suggests that factors beyond smoking behavior may contribute to increased risk of lung cancer among Black patients.

Social contextual factors play a role in differential lung cancer risk. Chronic exposure to social stressors, including neighborhood crime and violence, could contribute to lung cancer disparities (6). One of the mechanisms linking social conditions and lung cancer disparity may be stress responses. Chronic stress exposure has been shown to be associated with biophysical changes leading to an elevated risk of cancer development and progression (7). Allostatic load, defined as the cumulative burden of chronic stress, is elevated in residents of disadvantaged neighborhoods (8) and is associated with a 43% higher all-cause mortality in metastatic lung cancer patients (9). Exposure to chronic stress disrupts the regulation of circadian glucocorticoid secretion and clearance from circulation, leading to persistently elevated levels of cortisol (10). The glucocorticoid receptor (GR), a member of the nuclear receptor superfamily of transcription factors, is activated in response to glucocorticoids. The GR plays a crucial role in various aspects of normal lung development and the immune system (11). Moreover, GR levels vary across different tumor types and GR can act as a tumor activator or repressor based on the cellular context (12). In the presence of a ligand, the GR translocates to the nucleus, binding to glucocorticoid response elements on DNA. This binding generates nucleation sites for other coregulators and transcriptional proteins. Subsequently, a comprehensive transcriptional program is activated, which either increases or decreases expression levels of GR-responsive genes.

Our preliminary data indicate that, in the city of Chicago, residing in areas with high violent crime, particularly homicides, was associated with an increased risk of developing lung cancer by 31%, even after controlling for individual-level risk factors such as smoking history, gender, and age (13). Given the prevailing racial residential segregation and the spatial patterns of concentrated violent crime in Chicago, elevated lung cancer risk is notably clustered in Black communities on the west and south sides of the city. In this study, we examine whether differential exposure to neighborhood violent crime between Black and White residents may account for racial disparities in gene expression patterns related to lung cancer (14). We explore the association between neighborhood violent crime rates and gene expression in lung tumors. We further examine the variations in GR activity in tumors by the violent crime rate. Our analysis aims to establish a mechanistic connection between exposure to neighborhood violence and racial disparities in lung cancer, suggesting that tumor GR serves as the primary sensor and mediator of the associated tumor biology.

## Materials and Methods

### Samples

This study protocol has been reviewed and approved by the University of Illinois Chicago (UIC) Institutional Review Board (IRB# 2023-0726). We utilized 15 fresh frozen lung tumor samples and corresponding healthy adjacent lung tissue samples for this analysis. All samples were from the UIC Biorepository. Samples were obtained from patients who received care at the University of Illinois Hospital and resided in Chicago at the time of diagnosis and tissue collection. The samples were collected from lung cancer cases diagnosed between 2015 and 2021. Age, race, gender, residential zip code, and smoking status were retrieved from the electronic medical record by a designated honest broker.

Using patients’ residential zip codes, we appended violent crime rates (Table 1). Violent crime includes rape, robbery, aggravated assault, and homicide (15, 16). Violent crime rates and other zip code level measures were obtained from the Chicago Health Atlas and CrimeGrade.org (17). Using the zip code–level violent crime counts and the population size from the Chicago Health Atlas, the mean violent crime rates were calculated for the period of 2015 and 2021.

**Table 1:** Patient demographics

Characteristics	High-crime neighborhood (*n* = 5)	Medium-crime neighborhood (*n* = 4)	Low-crime neighborhood (*n* = 6)
Male (%)	3 (60)	2 (50)	4 (67)
Black (%)	4 (80)	2 (50)	4 (67)
Smoker (%)	5 (100)	3 (75)	4 (67)
Age, mean (SD)	64 (4.9)	69 (9.0)	57 (5.5)

The Kruskal–Wallis test was used to compare being male, Black, smoker, and mean age. Asymptotic significance was presented. The mean age of the sample was 62 years; and the cases from neighborhoods with low violent crime rates were younger, with a mean age of 57 years. There were no other differences in the distribution of gender, race, and smoking history.

### Spatial transcriptomics analysis

Visium (10× Genomics) spatial transcriptomics libraries were constructed at the Carl R. Woese Institution for Genomic Biology Core Facility and the DNA Services laboratory of the Roy J Carver Biotechnology Center (CBC) at the University of Illinois Urbana-Champaign (UIUC). A detailed description of the library preparation is mentioned in the Supplementary Material. Spatially barcoded reads were prepared following the 10× Genomics protocol for further analyses. Demultiplexed reads were mapped to the human genome using SpaceRanger 2.0.0. based on Ensembl Release 106 and visualized using Seurat (R package). All 15 samples were merged and normalized using SCTransform. Using PyGeoda v.0.0.8, univariate local Moran’s I was examined to identify contiguous barcodes with significant localization of gene expression (i.e., significant local spatial association of gene expression). We then tested the significance of spatial association using unadjusted *P*-values at the two significance levels of 0.05 and 0.01 (18). Genes in each sample with 5% or more of their barcodes classified as significant spatial association by the local Moran’s I test were identified as localized genes. Hotspot analysis, a tool commonly used in geospatial studies to identify spatial clusters, was used to identify regions with above average gene expression values, which indicates greater than expected by pure spatial randomness. Each barcode is assigned a hotspot label based on the hotspot analysis results. Four clusters were generated, namely clusters 1 (Highest expression within the sample), cluster 2 (High expression compared to neighboring barcodes), cluster 3 (Low expression compared to neighboring barcodes), and cluster 4 (Lowest expression within the sample), by assigning barcodes with a hotspot label to a corresponding cluster. Differential expression tests were performed for each gene between one cluster and the remaining three clusters using the Seurat FindMarkers function with default settings. The default Wilcoxon rank-sum test was used to calculate the statistical significance of genes with a log fold change >0.1.

### CUT&RUN assay

CUT&RUN was performed using 15 tumor samples, including one replicate for tumor 8, with the CUT&RUN Assay Kit (Cell Signaling, #86652) in accordance with the provided protocol. Briefly, 1 to 2 mg of tissue per pulldown was used. Tumor samples were homogenized, and cells were washed and bound to activated Concanavalin A magnetic beads. The cells were permeabilized with Digitonin buffer, and the bead-cell complex was incubated with GR antibody overnight at 4°C on a rotator. The bead-cell-antibody complex was washed using Digitonin buffer and incubated with pAG-MNase solution for 1 hour at 4°C. The bead-cell-antibody complex was then washed with Digitonin buffer, the pAG-MNase was activated with calcium chloride, and the complex was incubated for 30 minutes at 4°C. Stop buffer was used to stop the reaction, and DNA fragments were released via incubation at 37°C for 10 minutes. Input samples were homogenized, DNA was extracted using the DNA extraction buffer, the cells were sonicated, and lysate was clarified via centrifugation. For DNA purification and concentration, a DNA Clean and Concentrator Kit was used (Zymo Research, #D5205) to elute 12 μL. Antibodies used were: GR/NR3C1/Glucocorticoid Receptor, sc-393232, Santa Cruz (RRID:AB_2687823).

### CUT&RUN data analysis

CUT&RUN libraries were prepared by the High-Throughput Sequencing and Genotyping Unit in the CBC at UIUC. Paired-end FASTQ files were aligned to human reference (GRCh38) using Bowtie2 (v2.4.5), and BAM files were prepared using SAMtools (v1.17). Peak calling was performed using MACS2 (v2.2.5) for individual tumor sample reads with corresponding normal sample reads as the control. Narrow peak calling was performed for GR with a *P*-value cutoff and FDR of 0.001. Bed files were generated by filtering out binding regions found in the input, with the exception of tumor-specific and normal-specific bed files. Tumor-specific bed files were generated by filtering out binding regions also found in healthy tissue bed files. Normal-specific bed files were similarly generated by removing binding regions found in tumor bed files.

Output bam files were converted to bigwig format by Deeptools 3.5.2 (RRID:SCR_016366) bamCoverage. sBAM files were generated using samtools 1.15.1. (RRID:SCR_002105). Peaks were visualized using the UCSC Genome Browser (RRID:SCR_005780; ref. 19). Heatmaps and underlying values were generated using Deeptools 3.5.2 computeMatrix and plotHeatmaps (20). Gene annotation was performed using GREAT version 4.0.4 (21, 22), and binding site distribution was visualized using Bioconductor ChIPseeker 1.28.3 (RRID:SCR_006442; ref. 23). Motif analysis was performed using Homer v4.11 (24). Functional annotation of genes was performed in DAVID 2021 (RRID:SCR_001881) using Biocarta, Kegg, and Wikipathways (25, 26).

### Data availability

Raw data are available through the NIH GEO repository (GSE268048 and GSE268049).

## Results

### Gene expression programs and neighborhood violent crime rates in lung tumors

**Figure 1:**
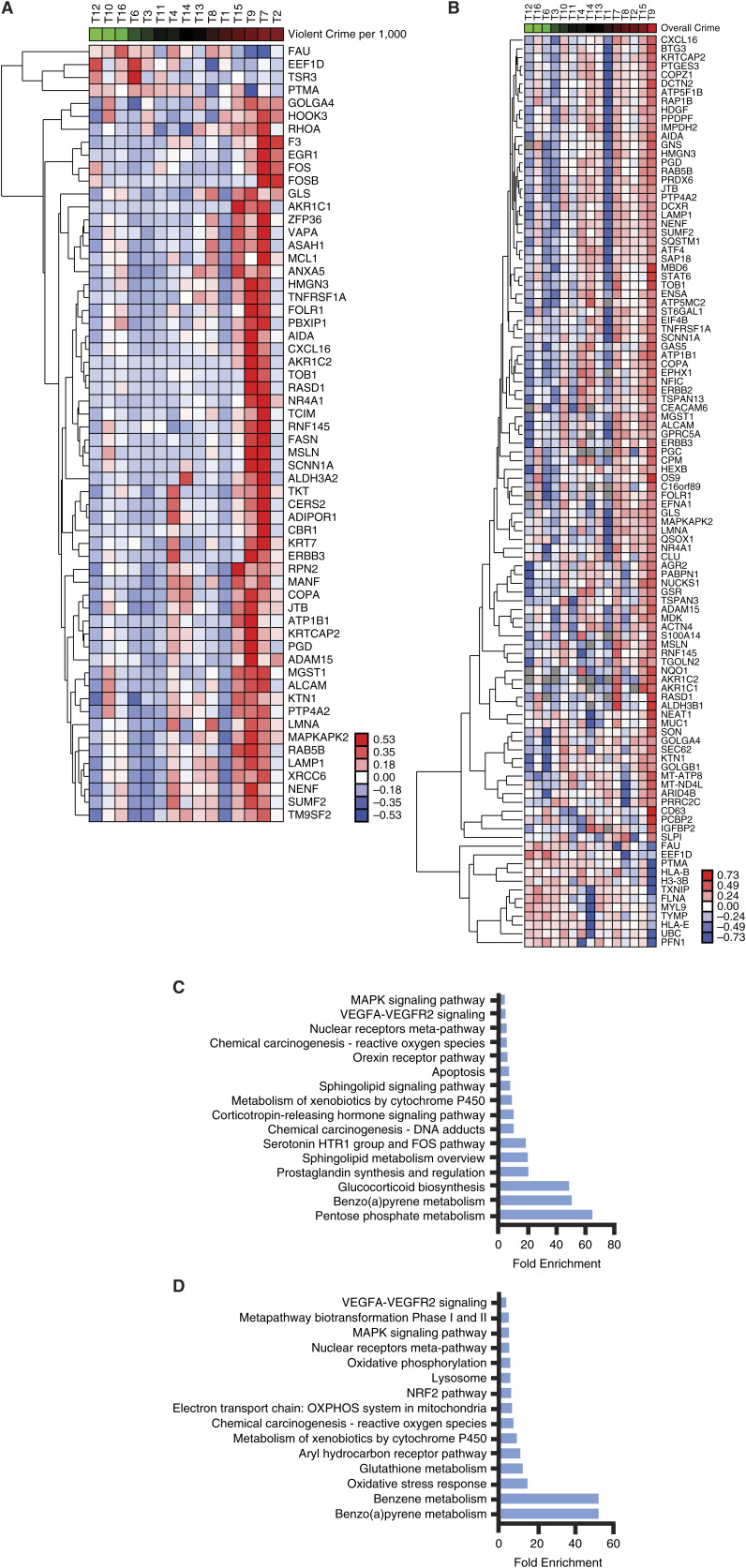
Gene expression programs and neighborhood violent crime rates in lung tumors. **A,** Heatmap of genes correlated with violent crime rates per 1,000 (*P* < 0.05). Average normalized gene expressions per tumor sample were extracted from spatial transcriptomics data and checked for correlation with violent crime per 1,000. Housekeeping genes were removed and data were log-transformed and hierarchically clustered prior to visualization. From (left to right), samples are ordered from lowest violent crime ranking to highest violent crime ranking. **B,** Heatmap of genes correlated with overall crime rates (*P* < 0.05). Average normalized gene expressions per sample were extracted from spatial transcriptomics data and checked for correlation with violent crime per 1,000. Housekeeping genes were removed and data was log-transformed and hierarchically clustered prior to visualization. From (left to right), samples are ordered from lowest violent crime ranking to highest violent crime ranking. **C,** Pathway analysis of genes upregulated with violent crime rates per 1,000 (*P* < 0.05). Upregulated genes were extracted from the violent crime heatmap, and functional annotation of genes was performed in DAVID using Biocarta, Kegg, and Wikipathways analysis. **D,** Pathway analysis of genes upregulated with overall crime (*P* < 0.05). Upregulated genes were extracted from the violent crime heatmap, and functional annotation of genes was performed in DAVID using Biocarta, Kegg, and Wikipathways analysis. Abbreviation. T, tumor.

Based on our previous findings that showed a 31% increase in lung cancer risk among those residing in areas with high violent crime (13), we identified genes correlated with violent crime rates with Pearson’s correlation (*P* < 0.05) using normalized counts obtained from the R package Seurat. Average gene expressions per tumor sample were correlated with various neighborhood factors, including violent crime rate, overall crime rate, % poverty, and racial/ethnic composition (% Black, % White, and % Hispanic residents). Heatmap visualizations of the results from correlation analyses showed distinct differences in genes upregulated and downregulated in samples from patients living in neighborhoods with high violent crime rates (Fig. 1A) and high overall crime rates (Fig. 1B). Correlation coefficients and *P*-values for genes correlated with the violent crime rate can be found in Supplementary Table S1. A pathway analysis of genes that were upregulated in relation to exposure to violent crime demonstrated an increase in stress hormone associated pathways. These included glucocorticoid biosynthesis and corticotropin-releasing hormone (CRH) signaling, which were not observed in the pathway analysis of genes upregulated in relation to the overall crime rates (Fig. 1C). In contrast, pathway analysis of genes upregulated in correlation with the overall violence rate showed a noticeable increase in pathways associated with environmental toxicant exposure, such as benzene metabolism, aryl hydrocarbon receptor signaling, and oxidative stress response, which were not seen in violent crime pathway analysis (Fig. 1D). Of note, we did not detect any significant correlation between gene expression levels of biologically significant pathways from Fig. 1 and neighborhood racial/ethnic composition (Supplementary Fig. S1A–S1C), % poverty (Supplementary Fig. S1D), or air pollution level (Supplementary Fig. S1E).

### Spatial genomics uncovers hotspots of transcriptomic changes associated with glucocorticoid receptor signaling

**Figure 2:**
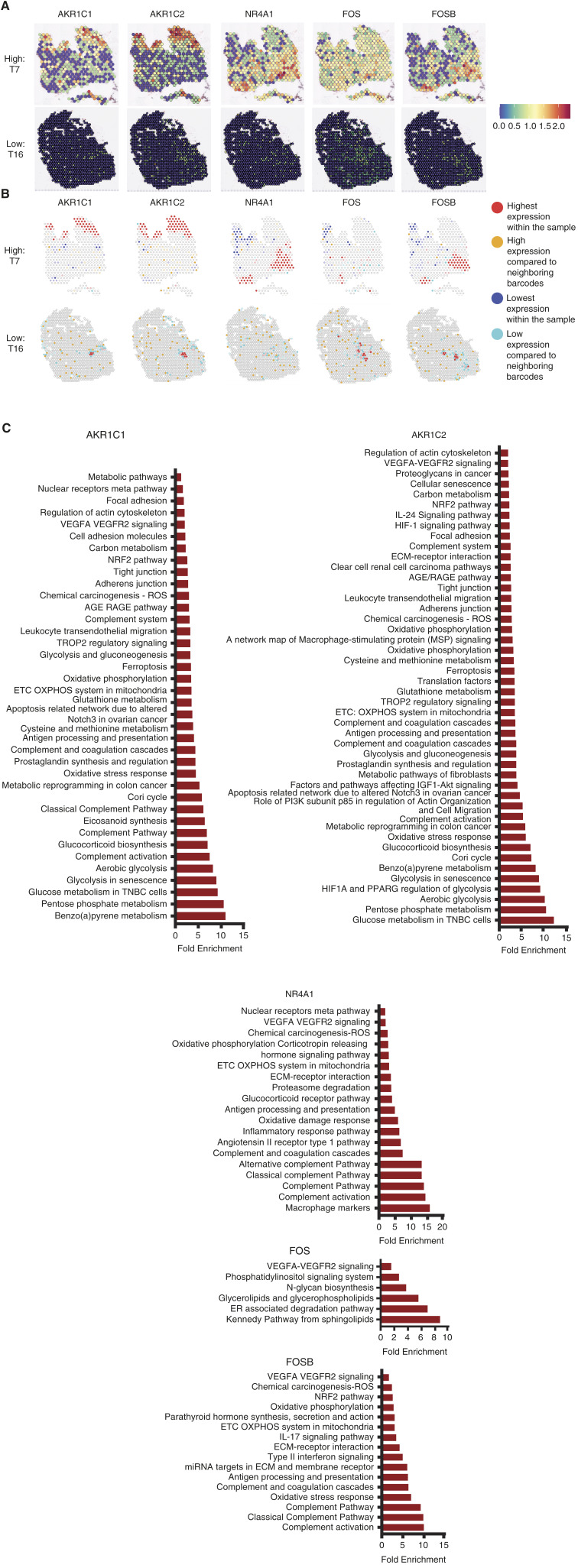
Spatial genomics uncovers hotspots of transcriptomic changes associated with glucocorticoid receptor signaling. **A,** Visualization of spatial gene expression of key genes correlated with the neighborhood violent crime rate in representative tumor samples from high and low-violence neighborhoods. Spatial expression was visualized in Seurat. **B,** Hot spot analysis of key genes correlated with the violent crime rate in representative tumor samples from high and low-violence neighborhoods. Hotspots were identified using the univariate local Moran’s test. Darker colors denote a *P*-value of <0.01 and lighter colors indicate a *P*-value of <0.05. **C,** Pathway analysis of genes differentially expressed within *P* < 0.01 highest expression within the sample hotspots. Differential gene expression analysis was performed using FindMarkers in Seurat, and genes with a non-adjusted *P*-value of <0.05 were used for pathway analysis. Analysis of pathways with a *P*-value of <0.05 was performed in DAVID using Biocarta, Kegg, and Wikipathways analysis.

Because we observed upregulation of glucocorticoid biosynthesis in the pathway analysis of genes, specifically those amplified in samples from high-violence neighborhoods (Fig. 1C), and considering the recognized role of GR in regulating gene expression under stress conditions, we hypothesized that genes associated with the neighborhood violent crime rate may exhibit unique GR-mediated gene regulation patterns associated with a more aggressive tumor phenotype. To validate this hypothesis, we further investigated genes from Fig. 1A that exhibited GR binding in our CUT&RUN data set that were also involved in stress response or tumor biology, as determined by their presence in pathways associated with cortisol signaling and tumorigenesis from Fig. 1C. We further narrowed our gene list of interest by selecting genes with elevated expression levels in samples from high-violence neighborhoods and low expression levels in samples from low-violence neighborhoods (Fig. 2A). Hotspot analysis identified spatial clusters of high violence and several of these genes of interest (Fig. 2B). These hotspots were observed in samples from high-violence neighborhoods and were diminished or absent in samples from low-violence neighborhoods.

To validate GR recruitment to chromatin within these genes, we utilized BigWig files from a previously published GR ChIP-Seq dataset and visualized GR peaks in proximity to the transcription start sites of these genes in a well-established lung cancer cell line, A549 cells (Supplementary Fig. S2; ref. 27). Notably, distinct peaks were observed near the transcription start site of all of these genes, suggesting that GR may act as a potential sensor and mediator of biological effects of neighborhood violent crime and regulator of genes to drive worsened tumor outcomes.

To further investigate if GR signaling was altered in these hotspots, we performed differential gene expression analysis to determine genes overexpressed in the clusters observed in Fig. 2B. Using the gene list expressed in these hotspots within the high-violence tumor samples, functional enrichment analysis revealed an upregulation of glucocorticoid biosynthesis in *AKR1C1*, *AKR1C2*, and *NR4A1* overexpression, indicating altered cortisol synthesis. We also observed a clear dysregulation of the inflammatory, immune response, antioxidant, proliferative, and angiogenic pathways in *AKR1C1*, *AKR1C2*, *NR4A1*, and *FOSB* hotspots (Fig. 2C). Pathway analysis of *FOS* overexpression hotspots in high-violence tumor samples found pathways associated with angiogenesis, the cell membrane, and sphingolipid metabolism. The hotspot analysis result further suggested altered cortisol metabolism and increased tumor aggressiveness in samples from patients residing in high-violence neighborhoods.

### GR-binding site distribution is altered in response to neighborhood violent crime

**Figure 3:**
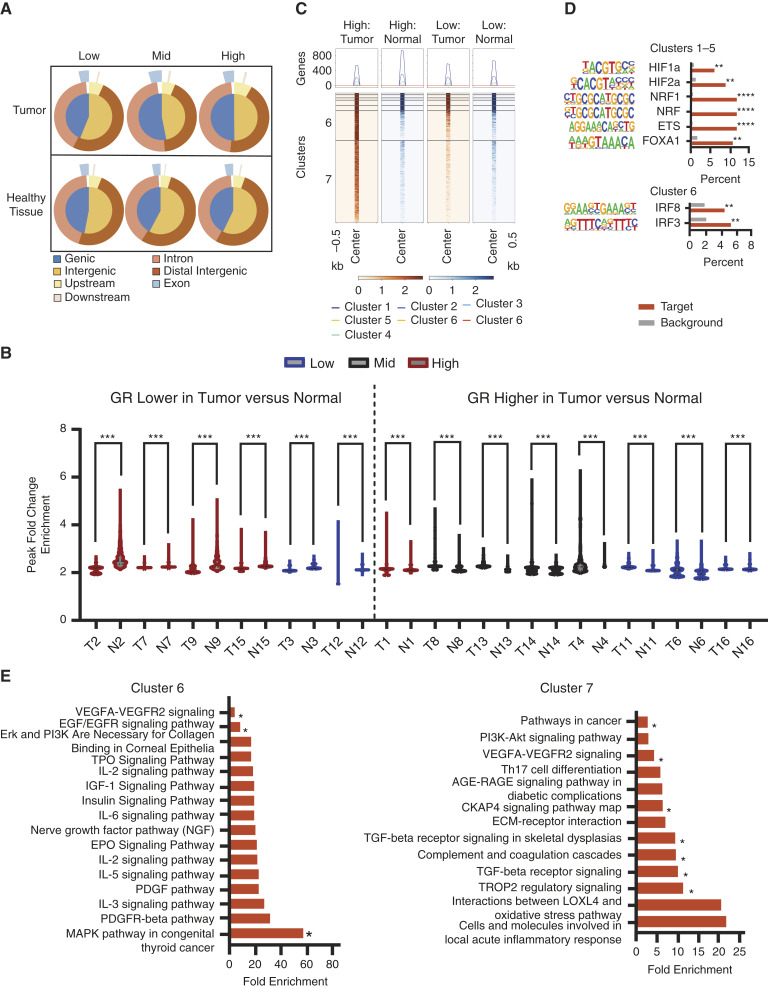
Distinct pathways altered within the GR cistrome in response to violent crime. **A,** Binding site distribution of GR in tumor samples and healthy tissue samples by low, mid, and high exposure to violence. Bam files for individual samples were merged by exposure to violence grouping. Merged tumor and merged normal bed files were generated relative to merged input files. Binding site distribution from these merged bed files was generated using ChIPSeeker. **B,** Comparison of enriched GR binding sites in tumor compared to healthy tissue samples. For each sample, tumor and healthy tissue bed files were generated by filtering out binding sites observed in the input. For each patient, the top 10% of enriched binding sites for tumor and healthy tissue were plotted and compared using the Kolmogorov–Smirnov test. A cutoff of 10% was selected to ensure a high confidence level in GR binding sites, as the top 10% of binding sites exhibited a high magnitude of GR binding. **C,** Comparison of the magnitude of GR binding sites from high-violence tumor samples in tumor vs. normal samples in high-violence vs. low-violence samples. BAM files for individual samples were merged by exposure to violence grouping. A merged bed file of high-violence tumor sample binding sites was generated relative to input binding sites. Merged sBAM files were generated using merged BAM files and the merged bed file. Heatmaps visualizing the magnitude of GR recruitment to chromatin were generated using Deeptools, and clusters were generated using the kmeans clustering algorithm. Darker shading indicates an increased magnitude of GR binding. **D,** Motif analysis of binding sites in clusters 1 to 5 and cluster 6 from (**C**). Motif analysis was performed using Homer. **E,** Pathway analysis of annotated genes from clusters 6 and 7 visualized in (**C**). Genes were annotated using GREAT analysis, and pathway analysis was performed in DAVID using Biocarta, Kegg, and Wikipathways analysis. *, *P* < 0.05; **, *P* < 0.01; ***, *P* < 0.0001; ****, *P* < 0.000001. Abbreviations. T, tumor; N, normal; Low, low exposure to violence; Mid, mid exposure to violence; High, high exposure to violence.

Due to the observed enrichment of genes associated with GR signaling (Fig. 1) and GR recruitment to regulatory regions of genes related to the neighborhood violent crime rate in lung cancer cell lines (Supplementary Fig. S2), we hypothesized that exposure to neighborhood violence influences GR activity by changing the recruitment pattern to chromatin in lung tumors. We employed CUT&RUN analysis using the tumor samples from Fig 1 as well as adjacent normal lung tissue from the same individuals to examine variations in GR recruitment to chromatin amid exposure to neighborhood violence. We visualized the distribution binding sites derived from CUT&RUN of lung tumors and corresponding healthy tissue samples. The distribution of GR enrichment varied by the violent crime rate. In high-violence exposure tumor samples, GR binding occurred in upstream binding sites, while low and mid-violence exposure tumor samples did not show such upstream binding (Fig. 3A). GR binding within healthy tissue samples displayed patterns more akin to low-violence exposure tumor samples than high-violence exposure samples (Fig. 3A).

We further explored the difference in GR binding enrichment between tumor and healthy tissues. The top 10% of peak fold change enrichment in each tumor sample was compared with its corresponding healthy tissue sample (Fig. 3B). Total number of binding sites is available in Supplementary Table S2. Exposure to neighborhood violent crime presented distinct differences in the magnitude of GR binding between tumor and healthy tissue samples. Of the five samples with exposure to high violence, four exhibited lower GR binding in their tumors compared to their normal tissue samples. For the two other samples with lower GR binding in tumor than normal tissues, one was from a low-violence but high-poverty neighborhood. All but one of the patients that exhibited higher GR binding in their tumor compared to the normal samples were from mid or low violence neighborhoods.

Next, we compared the magnitude of GR binding enrichment in tumor samples from patients in low compared to high-violence neighborhoods (Supplementary Fig. S3A). The top 10% enriched tumor-specific binding regions were compared between tumor samples, showing increased GR binding in tumor samples from high compared to low exposure to violent crime. A similar analysis of cancer-free tissue-specific binding regions revealed higher GR binding in healthy tissue samples from high compared to low exposure to violence (Supplementary Fig. S3B).

### Distinct pathways altered within the GR cistrome in response to neighborhood violence

Given the altered distribution of GR-binding sites and the number observed in tumor samples from high-violence neighborhoods (Fig. 3), as well as our previous finding documenting a 31% increase in lung cancer risk for individuals residing in areas with high crime (14), we hypothesized that the rewiring of the GR cistrome in response to neighborhood violent crime increases pathways associated with tumor aggressiveness. Using GR-binding sites found in tumors from high-violence neighborhoods, we visualized these binding sites in tumor and healthy tissue samples from patients living in high and low violence areas. While some overlapping GR binding sites were observed in clusters 1 through 5, we found two GR-binding clusters, clusters 6 and 7, with a greater magnitude of GR binding in tumor samples from high-violence neighborhoods (Fig. 3C). Motif analysis of these clusters showed distinct coregulation patterns. Motifs present in cluster 6 showed inflammation-specific GR coregulators IRF8 and IRF3 (28), while motifs in the overlapping GR-binding sites were those of GR coregulators HIF1-a, ETS, and FOXA1 (29, 30). Motif analysis of cluster 7 revealed no motifs with a high % target to % background ratio (Fig. 3D).

To determine the genes differentially bound by GR in tumor samples from high-violence neighborhoods, we examined pathways associated with clusters 6 and 7. Genes in each cluster were annotated, and pathway analysis was performed on genes found to have greater than 10% expression in our spatial transcriptomics dataset. An increase in inflammatory pathways and cellular proliferation pathways was observed in cluster 6, while cluster 7 genes were those associated with immune response pathways (Fig. 3E). To validate GR recruitment to chromatin within these genes, we utilized BigWig files from a previously published GR ChIP-Seq dataset and visualized GR peaks in proximity to these genes in a well-established lung cancer cell line, A549 cells (25). GR bindings to key genes within statistically significant pathways are available in Supplementary Fig. S5.

We then investigated GR binding patterns specific to tumor samples from low-violence neighborhoods (Supplementary Fig. S4A). Cluster 7 revealed distinct GR recruitment patterns in the low violence exposure tumors. We mapped these binding sites to the closest genes, and further filtered the gene list based on gene expression values in tumors, keeping genes with greater than 10% expression in our spatial transcriptomics dataset. We then performed pathway analysis on these genes. Higher GR recruitment to genes specific to immune response was found, as well as increased recruitment to proliferative and angiogenic genes. However, the fold change enrichment of GR binding in genes associated with these pathways was lower than those observed in the same or similar pathways noted in Fig. 3E (Supplementary Tables S3–S5).

Due to the observed changes in GR binding in tumor samples, we compared GR binding patterns in healthy tissue with tumor samples from high-violence neighborhoods (Supplementary Fig. S4B). Pathway analysis of cluster 6, which showed increased GR recruitment specific to normal tissue from patients in high-violence neighborhoods, was performed (Supplementary Table S6). While some proliferative and invasive pathways were noted, their fold enrichment was lower than those seen in low-violence tumor samples and lower still in comparison to high-violence tumor samples. Immune response pathways were notably absent. An analysis of GR-binding patterns in healthy tissue from low-violence neighborhoods revealed similar patterns (Supplementary Fig. S4C and Table S7).

## Discussion

In this study, we described the changes in GR recruitment to chromatin and how they relate to the expression of genes in lung tumor samples from patients living in different neighborhoods in Chicago. Our results suggested that GR acted as an indicator of violence-related chronic stress, and repositioning GR to certain DNA regions drove gene expression programs that are associated with lung tumorigenesis.

We also saw decreased GR binding in tumors compared to healthy tissues in patients residing in high-violence neighborhoods and the increased expression of cortisol-metabolizing enzymes AKR1C1, AKR1C3, and CBR1. This finding suggests that exposure to neighborhood violent crime increases cortisol clearing within the lung tumor microenvironment. The expression of these cortisol-inactivating enzymes and CRH-signaling genes were correlated with elevated violent crime, while genes correlated with other neighborhood factors were not associated with the stress response. Hotspot analysis of key GR-bound genes revealed not only increased expression of these genes in high violence exposure tumors, but also overexpression of proliferative and inflammatory genes. These results suggest that the type of chronic stress triggered by residing in high-violence neighborhoods significantly alters cortisol metabolism and GR binding within the tumor microenvironment, ultimately driving an increase in pathways associated with tumor aggressiveness.

While cortisol signaling is typically known for its protective effects in lung cancer through reducing inflammation (12), some studies show that GR can increase cell proliferation and metastases through the upregulation of IGF1, TGF-β, WNT, and Hippo pathways (31–33). Our observed upregulation of IGF1, VEGFA, VEGFR2, and TGF- β receptor signaling in our GR-binding sites in tumors from high violence neighborhoods agrees with the latter studies, underscoring the importance of classic GR signaling within the lung. While GR is typically protective, exposure to specific stressors such as neighborhood violence may lead to a rewiring of GR activity within the lung tumor microenvironment, potentially explaining the conflicting findings in existing research on cortisol and GR signaling in lung cancer.

In addition to increased proliferative and angiogenic pathways, we also observed increased GR recruitment to pathways associated with inflammation and immune response. Under ideal conditions, GR provides anti-inflammatory properties through immunosuppressive functions (11, 34, 35). However, our results indicate that there is increased recruitment to genes associated with pro-inflammatory and dysregulated immune response among tumor samples from patients living in high-violence neighborhoods. This may be driven by the elevated interferon regulatory factor (IRF) motifs in gene clusters with higher GR binding among tumor samples from high-violence areas. IRF3 is known to compete with GR for binding to anti-inflammatory GRIP1 through decreasing GRIP1 activity, resulting in increased inflammation (28). The altered GR binding and activity within gene clusters specific to high-violence neighborhoods may be due to IRFs out-competing or rewiring the actions of GR. Another potential mechanism might be the tethering of GR to IRF proteins. Increased activation of IRF factors might increase interactions of GR with IRFs, thus leading to increased inflammatory gene responses in tumor samples from individuals residing in high-violence neighborhoods. Further mechanistic studies are needed to tease out these possibilities.

This rewiring of GR recruitment may be driven by other factors as well. TNF-a, an inflammatory cytokine, has been shown to reshape the GR nuclear cofactor profile, resulting in a significant alteration in GR-induced gene expression (36). Further, TNF-a and GR are known to work cooperatively to activate pro-inflammatory pathways in human lung cancer cell line A549s, despite the usual anti-inflammatory function of GR (37). However, the impacts of exposure to violent crime on the GR interactome and GR isoforms have yet to be explored. Further research regarding the underlying mechanisms driving this rewiring is crucial for understanding the role of cortisol and GR signaling in lung tumors, particularly among those living in chronically stressful neighborhood environments.

There are limitations to our study. First, we used a small number of tumor samples representing different zip codes. For the most part, due to the availability of fresh frozen sample tissues, we were not able to include a more representative number of cases for this analysis. This small number of cases limits our ability to generalize our findings. Further analysis with a larger sample of tumor tissues will validate our current study. Second, related to the small sample size, a relatively large geographic area unit at the zip code level was used, which may introduce uncertainty in characterizing violence exposure at the individual level. Although zip code is a relatively large geographic unit, thus potentially large heterogeneity, zip code level neighborhood characteristics have been widely used in public health research as a valid area unit of analysis (38–40).

## Conclusion

Lung tumor samples from high-violence neighborhoods showed distinct GR cistrome and transcriptome profiles. We observed an upregulation of pathways associated with tumor aggressiveness, suggesting rewiring of GR binding and regulation driven by exposure to neighborhood violence. An increased understanding of the link between exposure to neighborhood violent crime, glucocorticoid signaling, and lung tumorigenesis could lead to improved screening criteria for people living in high-stress environments and consequently reduce racial disparities in lung cancer. Currently, the U.S. Preventive Services Task Force lung cancer screening guideline indicates that individuals aged 50 to 80 years who have a 20-pack-year smoking history are eligible for low-dose computed tomography every year. Due to racial residential segregation, Black Americans often reside in disadvantaged neighborhoods, thus, are disproportionately exposed to social stress, which may increase the risk of lung cancer through GR recruitment to binding sites associated with tumorigenesis. Incorporating neighborhood context, particularly violent crime, into the current lung cancer screening guideline could improve the early detection of lung cancer, thus reducing racial disparities in lung cancer outcomes.

Reducing the racial gap in exposure to violence in different communities would require policies that aim to achieve social and economic equity, such as resource reallocation, fair community investment, and disrupting crime and violence in the most disadvantaged neighborhoods in our cities. Ultimately, social policies promoting equitable neighborhood development are necessary to achieve health equity.

## Supplementary Material

Supplementary Materials and Methods Supplementary methods, expanded version of sequencing methodology.

Supplementary Figure S1 Heatmap and pathway analysis of genes correlated with neighborhood factors.

Supplementary Figure S2 GR recruitment to chromatin in key genes correlated with neighborhood violence.

Supplementary Figure S3 Magnitude of GR binding enrichment in tumor and normal tissue samples from patients in low compared to high-violence neighborhoods.

Supplementary Figure S4 Comparison of magnitude of GR binding sites in tumor vs. normal samples in high violence vs. low violence samples.

Supplementary Figure S5 GR recruitment to chromatin in key genes within statistically significant pathway from Figure 3E.

Supplementary Data Supplementary Figure Legends

Supplementary Table S1 Genes correlated with exposure to neighborhood violence.

Supplementary Table S2 Total binding site number in tumor and normal tissue samples.

Supplementary Table S3 Pathway analysis results of cluster 6 genes from Figure 4A.

Supplementary Table S4 Pathway analysis results of cluster 7 genes from Figure 4A.

Supplementary Table S5 Pathway analysis results of cluster 7 genes from Supplementary Figure 4A.

Supplementary Table S6 Pathway analysis results of cluster 6 genes from Supplementary Figure 4B.

Supplementary Table S7 Pathway analysis results of cluster 6 genes from Supplementary Figure 4C.

## References

[1] Regierer AC , Wolters R, Ufen MP, Weigel A, Novopashenny I, Köhne CH, . An internally and externally validated prognostic score for metastatic breast cancer: analysis of 2269 patients. Ann Oncol 2014;25:633–8. 24368402 10.1093/annonc/mdt539 PMC4433507

[2] Trinidad DR , Gilpin EA, Lee L, Pierce JP. Do the majority of Asian-American and African-American smokers start as adults? Am J Prev Med 2004;26:156–8. 14751329 10.1016/j.amepre.2003.10.008

[3] Rugo HS , Rumble RB, Macrae E, Barton DL, Connolly HK, Dickler MN, . Endocrine therapy for hormone receptor–positive metastatic breast cancer: American society of clinical oncology guideline. J Clin Oncol 2016;34:3069–103. 27217461 10.1200/JCO.2016.67.1487

[4] DeSantis CE , Siegel RL, Sauer AG, Miller KD, Fedewa SA, Alcaraz KI, . Cancer statistics for African Americans, 2016: progress and opportunities in reducing racial disparities. CA Cancer J Clin 2016;66:290–308. 26910411 10.3322/caac.21340

[5] Aldrich MC , Mercaldo SF, Sandler KL, Blot WJ, Grogan EL, Blume JD. Evaluation of USPSTF lung cancer screening guidelines among African American adult smokers. JAMA Oncol 2019;5:1318–24. 31246249 10.1001/jamaoncol.2019.1402 PMC6604090

[6] Shonkoff JP , Garner AS; Committee on Psychosocial Aspects of Child and Family Health; Committee on Early Childhood, Adoption, and Dependent Care; Section on Developmental and Behavioral Pediatrics. The lifelong effects of early childhood adversity and toxic stress. Pediatrics 2012;129:e232–246. 22201156 10.1542/peds.2011-2663

[7] Hanahan D , Weinberg RA. Hallmarks of cancer: the next generation. Cell 2011;144:646–74. 21376230 10.1016/j.cell.2011.02.013

[8] Ribeiro AI , Amaro J, Lisi C, Fraga S. Neighborhood socioeconomic deprivation and allostatic load: a scoping Review. Int J Environ Res Public Health 2018;15:1092. 29843403 10.3390/ijerph15061092 PMC6024893

[9] Obeng-Gyasi S , Li Y, Carson WE, Reisenger S, Presley CJ, Shields PG, . Association of allostatic load with overall mortality among patients with metastatic non-small cell lung cancer. JAMA Netw Open 2022;5:e2221626. 35797043 10.1001/jamanetworkopen.2022.21626 PMC9264034

[10] Dickmeis T . Glucocorticoids and the circadian clock. J Endocrinol 2009;200:3–22. 18971218 10.1677/JOE-08-0415

[11] Kadmiel M , Cidlowski JA. Glucocorticoid receptor signaling in health and disease. Trends Pharmacol Sci 2013;34:518–30. 23953592 10.1016/j.tips.2013.07.003 PMC3951203

[12] Mayayo-Peralta I , Zwart W, Prekovic S. Duality of glucocorticoid action in cancer: tumor-suppressor or oncogene? Endocr Relat Cancer 2021;28:R157–R171. 33852423 10.1530/ERC-20-0489

[13] Kim SJ , Kery C, An J, Rineer J, Bobashev G, Matthews AK. Racial/ethnic disparities in exposure to neighborhood violence and lung cancer risk in Chicago. Soc Sci Med 2024;340:116448. 38043441 10.1016/j.socscimed.2023.116448 PMC10836639

[14] Conway EM , Pikor LA, Kung SH, Hamilton MJ, Lam S, Lam WL, . Macrophages, inflammation, and lung cancer. Am J Respir Crit Care Med 2016;193:116–30. 26583808 10.1164/rccm.201508-1545CI

[15] Federal Bureau of Investigation . Violent crime. Washington (DC): Federal Bureau of Investigation [cited 2023 Jul 30]. Available from: https://ucr.fbi.gov/crime-in-the-u.s/2010/crime-in-the-u.s.-2010/violent-crime.

[16] National Institute of Justice . Violent crime. Washington (DC): U.S. Department of Justice Office of Justice Programs 2023.

[17] Boukouris AE , Zervopoulos SD, Michelakis ED. Metabolic enzymes moonlighting in the nucleus: metabolic regulation of gene transcription. Trends Biochem Sci 2016;41:712–30. 27345518 10.1016/j.tibs.2016.05.013

[18] Anselin L , Li X, Koschinsky J. GeoDa, from the desktop to an ecosystem for exploring spatial data. Geographical Anal 2021;54:439–66.

[19] Kent WJ , Sugnet CW, Furey TS, Roskin KM, Pringle TH, Zahler AM, . The human genome browser at UCSC. Genome Res 2002;12:996–1006. 12045153 10.1101/gr.229102 PMC186604

[20] Ramírez F , Ryan DP, Grüning B, Bhardwaj V, Kilpert F, Richter AS, . deepTools2: a next generation web server for deep-sequencing data analysis. Nucleic Acids Res 2016;44:W160–5. 27079975 10.1093/nar/gkw257 PMC4987876

[21] McLean CY , Bristor D, Hiller M, Clarke SL, Schaar BT, Lowe CB, . GREAT improves functional interpretation of cis-regulatory regions. Nat Biotechnol 2010;28:495–501. 20436461 10.1038/nbt.1630 PMC4840234

[22] Tanigawa Y , Dyer ES, Bejerano G. WhichTF is functionally important in your open chromatin data? PLoS Comput Biol 2022;18:e1010378. 36040971 10.1371/journal.pcbi.1010378 PMC9426921

[23] Yu G , Wang LG, He QY. ChIPseeker: an R/Bioconductor package for ChIP peak annotation, comparison and visualization. Bioinformatics 2015;31:2382–3. 25765347 10.1093/bioinformatics/btv145

[24] Heinz S , Benner C, Spann N, Bertolino E, Lin YC, Laslo P, . Simple combinations of lineage-determining transcription factors prime cis-regulatory elements required for macrophage and B cell identities. Mol Cell 2010;38:576–89. 20513432 10.1016/j.molcel.2010.05.004 PMC2898526

[25] Huang DW , Sherman BT, Lempicki RA. Systematic and integrative analysis of large gene lists using DAVID bioinformatics resources. Nat Protoc 2009;4:44–57. 19131956 10.1038/nprot.2008.211

[26] Huang DW , Sherman BT, Lempicki RA. Bioinformatics enrichment tools: paths toward the comprehensive functional analysis of large gene lists. Nucleic Acids Res 2009;37:1–13. 19033363 10.1093/nar/gkn923 PMC2615629

[27] Gertz J , Savic D, Varley KE, Partridge EC, Safi A, Jain P, . Distinct properties of cell-type-specific and shared transcription factor binding sites. Mol Cell 2013;52:25–36. 24076218 10.1016/j.molcel.2013.08.037 PMC3811135

[28] Reily MM , Pantoja C, Hu X, Chinenov Y, Rogatsky I. The GRIP1:IRF3 interaction as a target for glucocorticoid receptor-mediated immunosuppression. EMBO J 2006;25:108–17. 16362036 10.1038/sj.emboj.7600919 PMC1356362

[29] Kodama T , Shimizu N, Yoshikawa N, Makino Y, Ouchida R, Okamoto K, . Role of the glucocorticoid receptor for regulation of hypoxia-dependent gene expression. J Biol Chem 2003;278:33384–91. 12810720 10.1074/jbc.M302581200

[30] Srivastava S , Nataraj NB, Sekar A, Ghosh S, Bornstein C, Drago-Garcia D, . ETS proteins bind with glucocorticoid receptors: relevance for treatment of Ewing sarcoma. Cell Rep 2019;29:104–17.e4. 31577941 10.1016/j.celrep.2019.08.088 PMC6899513

[31] Prekovic S , Schuurman K, Mayayo-Peralta I, Manjón AG, Buijs M, Yavuz S, . Glucocorticoid receptor triggers a reversible drug-tolerant dormancy state with acquired therapeutic vulnerabilities in lung cancer. Nat Commun 2021;12:4360. 34272384 10.1038/s41467-021-24537-3 PMC8285479

[32] Obradović MMS , Hamelin B, Manevski N, Couto JP, Sethi A, Coissieux MM, . Glucocorticoids promote breast cancer metastasis. Nature 2019;567:540–4. 30867597 10.1038/s41586-019-1019-4

[33] Perez Kerkvliet C , Dwyer AR, Diep CH, Oakley RH, Liddle C, Cidlowski JA, . Glucocorticoid receptors are required effectors of TGFβ1-induced p38 MAPK signaling to advanced cancer phenotypes in triple-negative breast cancer. Breast Cancer Res 2020;22:39. 32357907 10.1186/s13058-020-01277-8 PMC7193415

[34] Escoter-Torres L , Caratti G, Mechtidou A, Tuckermann J, Uhlenhaut NH, Vettorazzi S. Fighting the fire: mechanisms of inflammatory gene regulation by the glucocorticoid receptor. Front Immunol 2019;10:1859. 31440248 10.3389/fimmu.2019.01859 PMC6693390

[35] Ronchetti S , Migliorati G, Bruscoli S, Riccardi C. Defining the role of glucocorticoids in inflammation. Clin Sci (Lond) 2018;132:1529–43. 30065045 10.1042/CS20171505

[36] Dendoncker K , Timmermans S, Vandewalle J, Eggermont M, Lempiäinen J, Paakinaho V, . TNF-α inhibits glucocorticoid receptor-induced gene expression by reshaping the GR nuclear cofactor profile. Proc Natl Acad Sci U S A 2019;116:12942–51. 31182584 10.1073/pnas.1821565116 PMC6600915

[37] Hermoso MA , Matsuguchi T, Smoak K, Cidlowski JA. Glucocorticoids and tumor necrosis factor alpha cooperatively regulate toll-like receptor 2 gene expression. Mol Cell Biol 2004;24:4743–56. 15143169 10.1128/MCB.24.11.4743-4756.2004 PMC416411

[38] Kerry R , Goovaerts P, Haining RP, Ceccato V. Applying geostatistical analysis to crime data: car-related thefts in the Baltic states. Geogr Anal 2010;42:53–77. 22190762 10.1111/j.1538-4632.2010.00782.x PMC3242381

[39] Lin WC , Lin YP, Wang YC, Chang TK, Chiang LC. Assessing and mapping spatial associations among oral cancer mortality rates, concentrations of heavy metals in soil, and land use types based on multiple scale data. Int J Environ Res Public Health 2014;11:2148–68. 24566045 10.3390/ijerph110202148 PMC3945590

[40] Goovaerts P . Combining areal and point data in geostatistical interpolation: applications to soil science and medical geography. Math Geosci 2010;42:535–54. 21132098 10.1007/s11004-010-9286-5 PMC2995922

