# Supplementary material for: The Effect of Exposure to Neighborhood Violence on Glucocorticoid Receptor Signaling in Lung Tumors
Source: Cancer Res Commun. 2024 Jul 3;4(7):1643–54. doi: 10.1158/2767-9764.CRC-24-0032 (PMC11221527; doi:10.1158/2767-9764.CRC-24-0032)
Supplement: Supplementary Materials and Methods — Supplementary methods, expanded version of sequencing methodology. [file crc-24-0032_supplementary_materials_and_methods_suppsm.docx]

**Supplementary Materials**

**Supplementary Materials Index**

Spatial Transcriptomics Supplementary methods (Pages 2-5)

Sample Handling (Pages 2-3)

RNA Extraction for Quality Check (Pages 3-4)

10x Visium Tissue Optimization and cDNA extraction (Page 4)

Final construction and sequencing of 10x Visium libraries (Pages 4-5)

Spatial transcriptomics supplementary methods

*Sample Handling*

Sixteen 1mL cryo tubes were transferred to Cytometry and Microscopy to Omics (CMtO) facility on dry ice and placed in the -80ᵒC freezer. Each tube contained one small piece of lung tumor tissue. Optimum cutting temperature (OCT) was chilled in a -20ᵒC cryochamber associated with a cryostat (Cryostar, NX70, Epredia, Kalamazoo, MI). Each sample was removed from the -80ᵒC freezer and kept on dry ice. A small amount of OCT was placed in a cryomold, which was then placed on the dry ice. Using chilled forceps, the tissue was removed from the cryovial and placed on top of the OCT in this cryomold. While still on dry ice, each tissue mold was photographed next to the original cryovial to record the sample ID and size of the tissue. The tissue was then fully covered with the chilled OCT, left on dry ice until entirely frozen, then wrapped in aluminum foil and placed in a Ziplock bags with the original cryotube, and returned to the -80ᵒC freezer.

The images of each sample were reviewed and the largest sample, lung tumor #8, was selected to use for TO (10x Genomics Visium Tissue Optimization Protocol) to calculate the permeabilization time. Lung Tumor #8, a TO slide, and one 10x Genomics Gene Expression (GE) slide were placed in the cryochamber to equilibrate. All samples were sectioned at 10um thickness. The first tissue sections (all sections were collected) from lung tumor #8 were collected in a pre-chilled DNAse/RNAse free 1.5mL tube for or further characterization. Next, approximate middle of the sections approach sections was placed onto the TO slide, avoiding curls and folding as much as possible. A section from this tissue was also added to the pre-chilled GE slide, to prevent multiple temperature fluctuations from -80ᵒC to -20ᵒC. The three other samples for this GE slide were also equilibrated in the cryochamber. Sections from each sample were added to pre-chilled DNAse/RNAse free 1.5mL tubes until the sectioning face showed the sample’s maximum size based on prior images. These sections were then positioned on the slide avoiding curls and folds as much as possible, and the face of each tissue was covered with chilled OCT prior to being returned to the -80ᵒC freezer. The remaining 12 samples were handled the same way as above, with all samples and slides being returned to -80ᵒC as quickly as possible.

*RNA Extraction for Quality Check*

Additional sections were made on select representative samples/tissues for RNA integrity check with RNA integrity number (RIN). After tumor tissues were embedded in OCT, 10micro sections were collected in sterile 1.5ml centrifuge tubes and kept on dry ice and at -80C until provided to the DNA Services lab for RNA extraction. RNA was extracted from 2-3 OCT-embedded tumor tissues slices from each of 16 samples. Slices were put in a grinding tube (2305-2800SS, SPEX SamplePrep, NJ) and kept on dry ice before processing. RNA extraction was performed with the RNeasy Plus Micro Kit (Qiagen, MD). Briefly, 600ul of RTL buffer containing 40mM DTT (Bioworld, OH) was added to the grinding tube and vortexed for 30s, followed by bead-beating at speed 1500rpm for 10s on the 2010/GenoGrinder (SPEX SamplePrep, NJ). All lysate from each sample was transferred to gDNA Eliminator spin columns, centrifuged for 30s at 8,000xg. The flow-through was transferred into a new tube and an equal volume of 70% ethanol was added to the lysate and mixed by pipetting. All lysate was again transferred into an RNeasy MiniElute spin column and placed into a 2ml collection tube, centrifuged 15s at 8,000xg, and the flow through discarded. The column was washed consecutively with RW1, RPE, and 80% ethanol. A final centrifuge step for an additional 5 minutes into a new collection tube with the lid open, to remove all final ethanol, was performed and then purified RNA was eluted in 7ul nuclease-free water. The RNA was quantitated with Qubit high-sensitivity RNA reagent (Thermofisher, CA) and the integrity was evaluated in a Fragment Analyzer and Bioanalzyer (Agilent, CA).

*10x Visium Tissue Optimization and cDNA extraction*

For the TO slide, a permeabilization time of 18 minutes (after consultation with 10x Genomics technical support) was selected after following and processing 10X Genomics Visium Tissue Optimization User Guide. This processing includes staining and imaging for H and E using a Axiocam 712 color camera (Carl Zeiss, Oberkochen, Germany) followed by fluorescence imaging at the TRITC channel under a Hamamatsu Fusion HT black and while camera (Hamamatsu Photonics, Japan), in a LSM 980 Airyscan 2 super resolution microscope (Carl Zeiss, Oberkochen, Germany) housed in CMtO. The first two GE slides were processed for cDNA extraction on one day and the final two GE slides processed the following day. The GE slides were processed following the 10X Genomics Visium Gene Expression Reagent Kits protocol, Revision F with appropriate materials and associated tools. High resolution H and E images were obtained using the Axiocam 712 color cameral associated with the LSM 980 Airyscan 2 super resolution microscope (Carl Zeiss, Oberkochen, Germany) prior to permeabilization. The collected cDNA from each section is delivered to DNA services facility (Carver Biotech Center) located in the same building as soon as after completion of extraction.

*Final construction and sequencing of 10x Visium libraries*

Spatially-barcoded cDNAs were immediately brought to the DNA Services Core for amplification, strictly following the 10x Genomics Fresh FrozenVisium Spatial Gene Expression protocol. The amplified cDNAs were enzymatically fragmented and converted into sequencing-ready, dual-indexed libraries through standard end-repair, A-tailing, adaptor ligation, and PCR. The final libraries were quantitated on Qubit and the average size determined on the AATI Fragment Analyzer (Advanced Analytics, Ames, IA). The libraries were pooled by calculated capture spot coverage via Loupe Browser and the pool diluted to 5nM concentration, then further quantitated by qPCR on a Bio-Rad CFX Connect Real-Time System (Bio-Rad Laboratories, Inc. CA).  The final pool was sequenced on an Illumina NovaSeq 6000 to a length of 28nt (read 1, contains the spot barcode and unique molecular identifier used for removing PCR duplicates), 10nt for each index (libraries contain unique dual indexes to prevent index switching) and 150nt for read 2 (the cDNA read) to a minimum depth of at least 100,000 cDNA reads per spot.   Fastq.gz files were generated and demultiplexed with SpaceRanger 1.3.0.
