# Supplementary material for: The Effect of Exposure to Neighborhood Violence on Glucocorticoid Receptor Signaling in Lung Tumors
Source: Cancer Res Commun. 2024 Jul 3;4(7):1643–54. doi: 10.1158/2767-9764.CRC-24-0032 (PMC11221527; doi:10.1158/2767-9764.CRC-24-0032)
Supplement: Supplementary Figure S2 — GR recruitment to chromatin in key genes correlated with neighborhood violence. [file crc-24-0032_supplementary_figure_s2_suppsf2.pdf]

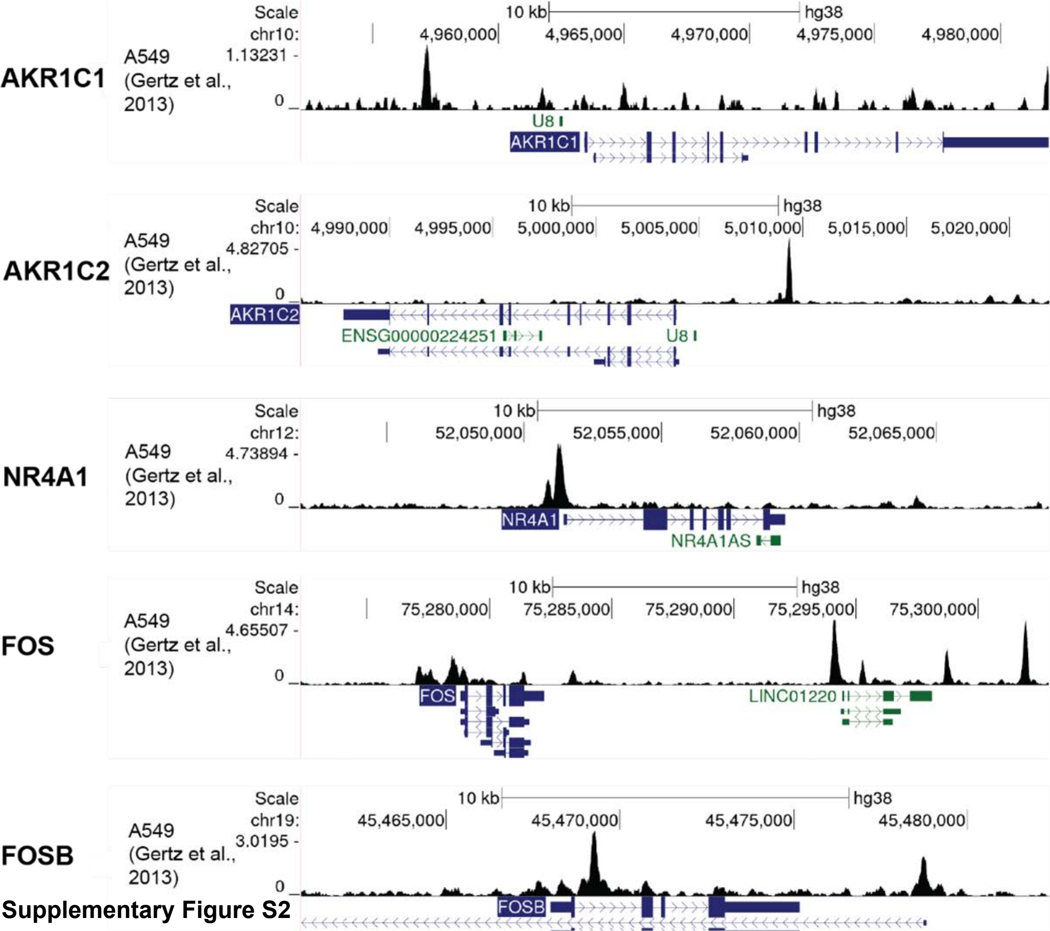

Supplementary Figure S2
