# Supplementary material for: The Effect of Exposure to Neighborhood Violence on Glucocorticoid Receptor Signaling in Lung Tumors
Source: Cancer Res Commun. 2024 Jul 3;4(7):1643–54. doi: 10.1158/2767-9764.CRC-24-0032 (PMC11221527; doi:10.1158/2767-9764.CRC-24-0032)

Supplementary Figure S3

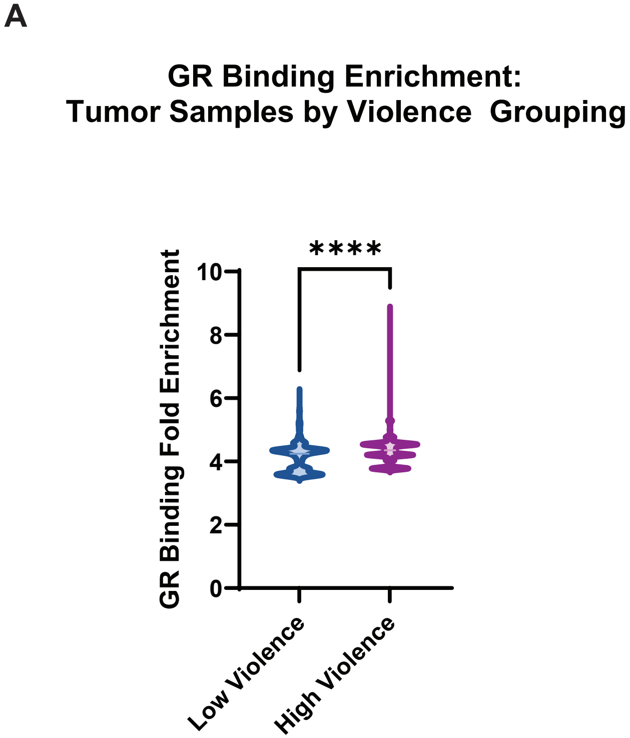

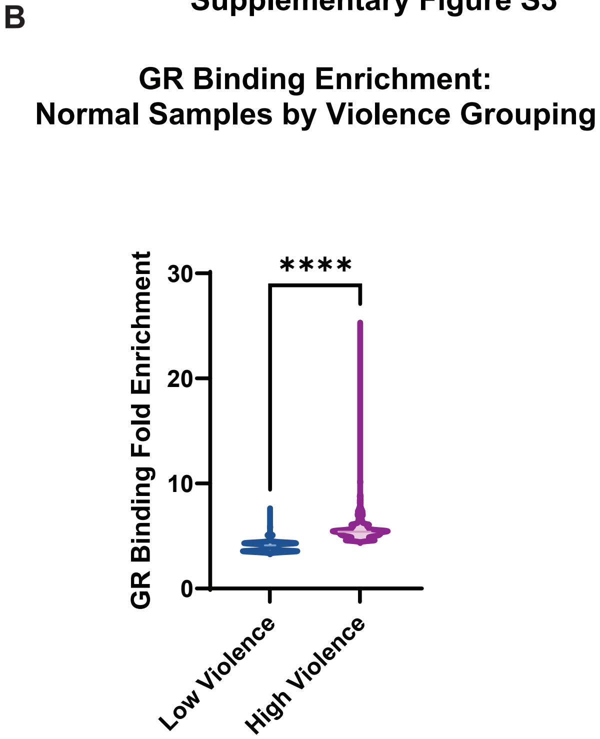

Supplement: Supplementary Figure S3 — Magnitude of GR binding enrichment in tumor and normal tissue samples from patients in low compared to high-violence neighborhoods. [file crc-24-0032_supplementary_figure_s3_suppsf3.pdf]
