# Supplementary figures and images for: The Effect of Exposure to Neighborhood Violence on Glucocorticoid Receptor Signaling in Lung Tumors
Source: Cancer Res Commun. 2024 Jul 3;4(7):1643–54. doi: 10.1158/2767-9764.CRC-24-0032 (PMC11221527; doi:10.1158/2767-9764.CRC-24-0032)

Supplementary Figure S4

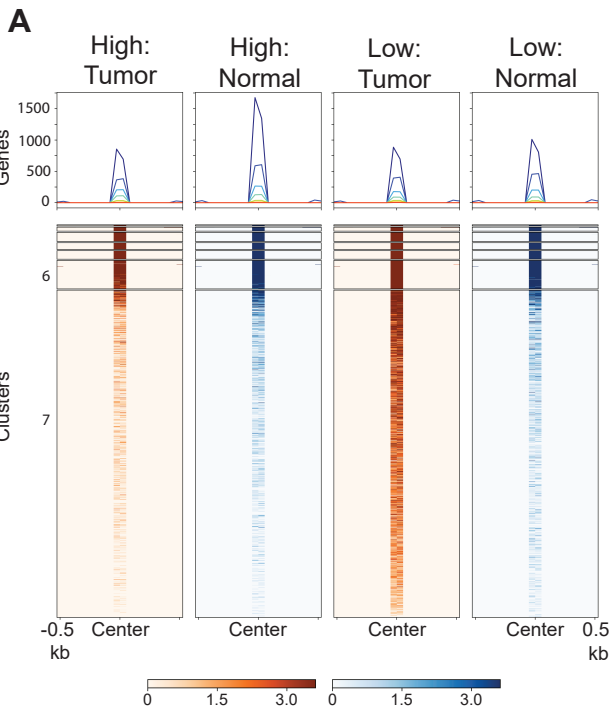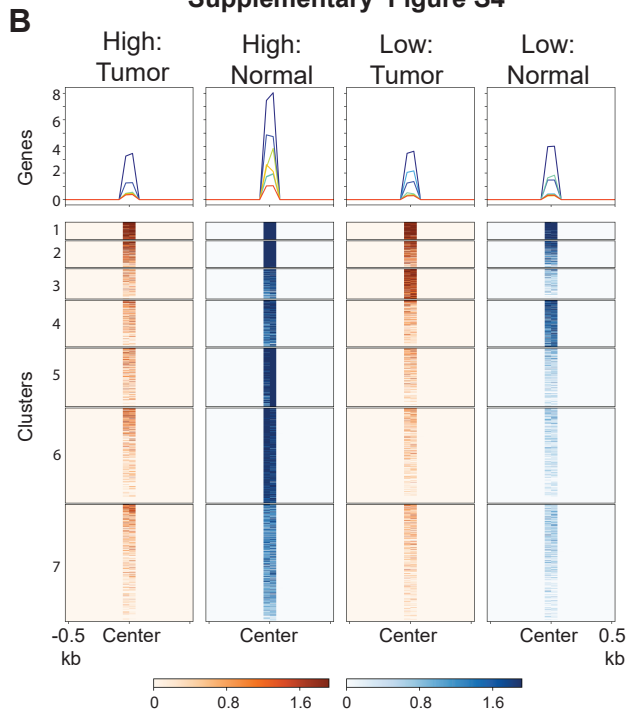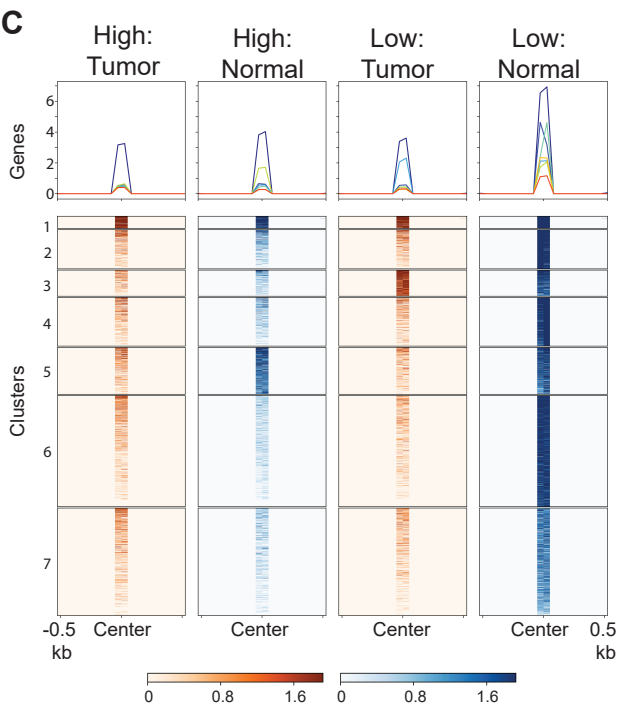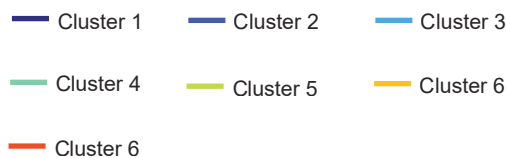

Supplement: Supplementary Figure S4 — Comparison of magnitude of GR binding sites in tumor vs. normal samples in high violence vs. low violence samples. [file crc-24-0032_supplementary_figure_s4_suppsf4.pdf]

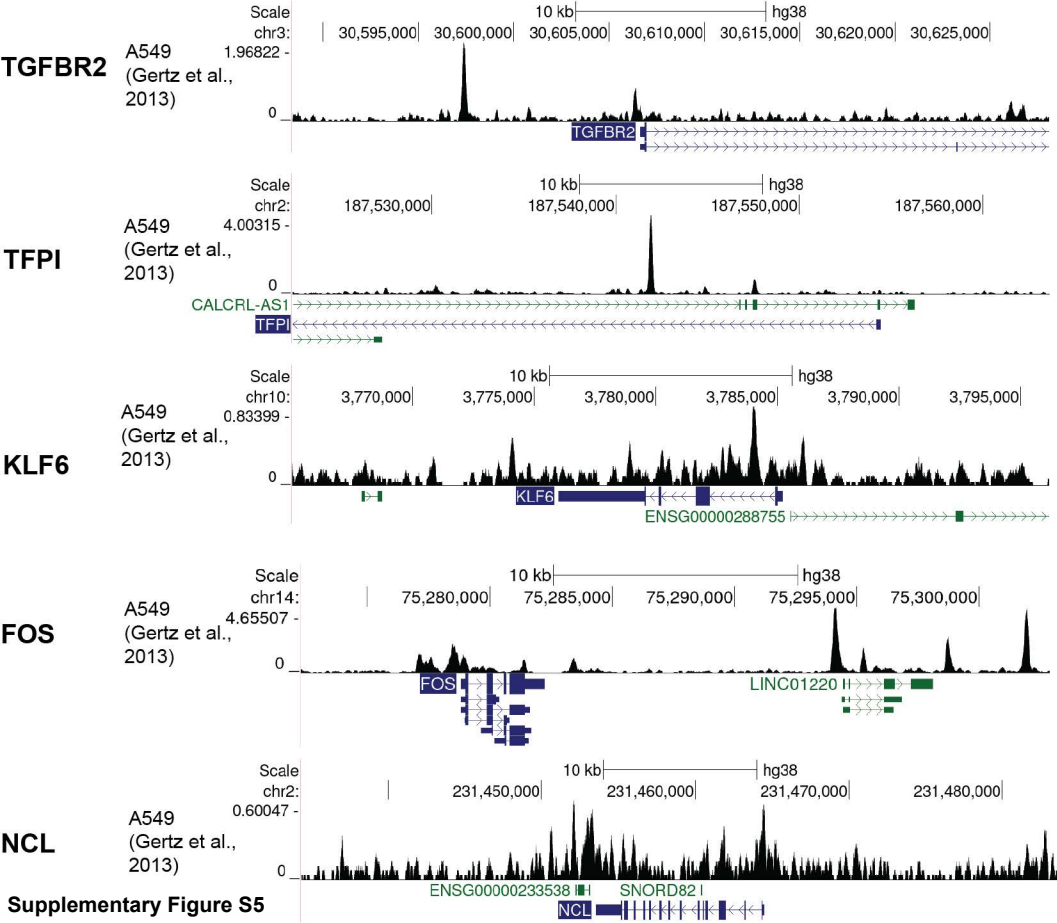

### Supplementary Figure S5

Supplement: Supplementary Figure S5 — GR recruitment to chromatin in key genes within statistically significant pathway from Figure 3E. [file crc-24-0032_supplementary_figure_s5_suppsf5.pdf]
