# Supplementary material for: The Effect of Exposure to Neighborhood Violence on Glucocorticoid Receptor Signaling in Lung Tumors
Source: Cancer Res Commun. 2024 Jul 3;4(7):1643–54. doi: 10.1158/2767-9764.CRC-24-0032 (PMC11221527; doi:10.1158/2767-9764.CRC-24-0032)
Supplement: Supplementary Data — Supplementary Figure Legends [file crc-24-0032_supplementary_data_suppsd.docx]

**Supplementary figure legends.**

**Supplementary Figure 1.** Heatmap and pathway analysis of genes correlated with neighborhood factors. Average gene expressions per sample were extracted from spatial transcriptomics data and correlated with violent crime per 1,000 (p<0.05). Housekeeping genes were removed and data were log transformed and hierarchically clustered prior to visualization. Genes were extracted from the heatmap, and functional annotation of genes was performed in DAVID using Biocarta, Kegg, and Wikipathways analysis (p<0.05).

1A. Heatmap and pathway analysis of genes correlated with % Black residents.

1B. Heatmap and pathway analysis of genes correlated with % Hispanic residents.

1C. Heatmap and pathway analysis of genes correlated with % White residents.

1D. Heatmap and pathway analysis of genes correlated with % neighborhood poverty.

1E. Heatmap of genes correlated with % of particulate matter 2.5.

Abbreviations. pm (particular matter)

**Supplementary Figure 2.** GR recruitment to chromatin in key genes correlated with neighborhood violence. Peaks from GR ChIP-seq performed in dexamethasone-treated A549 lung cancer cell lines from Gertz et al.(1) were visualized in the UCSC Genome Browser in AKR1C1, AKR1C2, NR4A1, FOS, and FOSB genes.

**Supplementary Figure 3.** Magnitude of GR binding enrichment in tumor and normal tissue samples from patients in low compared to high-violence neighborhoods.

3A. Magnitude of GR binding enrichment in tumor samples from patients in low compared to high-violence neighborhoods. For each sample, tumor bed files were generated via filtering out binding sites observed in normal tissue samples. The top 10% of enriched binding sites for each high violence tumor sample and each low violence tumor sample were plotted and compared using the Kolmogorov-Smirnov test.

3B. Magnitude of GR binding enrichment in normal tissue samples from patients in low compared to high-violence neighborhoods. For each sample, normal bed files were generated via filtering out binding sites observed in tumor samples. The top 10% of enriched binding sites for each high violence normal tissue sample and each low violence normal tissue sample were plotted and compared using the Kolmogorov-Smirnov test.

**Supplementary Figure 4.** Comparison of magnitude of GR binding sites in tumor vs. normal samples in high violence vs. low violence samples.

4A. Comparison of magnitude of GR binding sites from low violence tumor samples in tumor vs. normal samples in high violence vs. low violence samples. BAM files for individual samples were merged by exposure to violence grouping. A merged bed file of low violence tumor sample binding sites was generated. Merged sBAM files were generated using merged BAM files and the merged bed file. Heatmaps were generated using Deeptools.

4B. Comparison of magnitude of GR binding sites from high violence normal samples in tumor vs. normal samples in high violence vs. low violence samples. BAM files for individual samples were merged by exposure to violence grouping. A merged bed file of high violence normal tissue sample binding sites was generated. Merged sBAM files were generated using merged BAM files and the merged bed file. Heatmaps were generated using Deeptools.

4C. Comparison of magnitude of GR binding sites from low violence normal samples in tumor vs. normal samples in high violence vs. low violence samples. BAM files for individual samples were merged by exposure to violence grouping. A merged bed file of low violence normal sample binding sites was generated. Merged sBAM files were generated using merged BAM files and the merged bed file. Heatmaps were generated using Deeptools.

**Supplementary Figure 5.** GR recruitment to chromatin in key genes within statistically significant pathway from Figure 3E. Peaks from GR ChIP-seq performed in dexamethasone-treated A549 lung cancer cell lines from Gertz et al.(1) were visualized in the UCSC Genome Browser in TGFBR2 (from TGF beta signaling pathway), TFPI (from complement and coagulation cascades pathway), KLF6 (from TGF beta signaling pathway), FOS (from TGF beta signaling pathway), and NCL (from VEGFA VEGFR2 signaling pathway) genes.

**References**

1. Gertz J, Savic D, Varley KE, Partridge EC, Safi A, Jain P, Cooper GM, Reddy TE, Crawford GE, Myers RM. Distinct properties of cell-type-specific and shared transcription factor binding sites. *Mol Cell*. 2013;52(1):25-36.
