# Supplementary material for: The Effect of Exposure to Neighborhood Violence on Glucocorticoid Receptor Signaling in Lung Tumors
Source: Cancer Res Commun. 2024 Jul 3;4(7):1643–54. doi: 10.1158/2767-9764.CRC-24-0032 (PMC11221527; doi:10.1158/2767-9764.CRC-24-0032)
Supplement: Supplementary Table S1 — Genes correlated with exposure to neighborhood violence. [file crc-24-0032_supplementary_table_s1_suppst1.pdf]

Supplementary Table 1. Genes correlated with exposure to neighborhood violence.

Gene	Correlation coefficient	p-value
RPL18A	-0.80263	0.00032
F3	0.76745	0.00084
NR4A1	0.73667	0.00173
EGR1	0.7135	0.00282
RPL10A	-0.679	0.00538
GOLGA4	0.67129	0.00614
RPS10	-0.66292	0.00707
RPL13	-0.64896	0.00886
FAU	-0.64431	0.00952
ZFP36	0.63165	0.01154
RPS15A	-0.62188	0.01332
MGST1	0.61437	0.01482
COPA	0.61388	0.01492
TCIM	0.61381	0.01493
RHOA	0.61212	0.01529
HMGN3	0.61182	0.01536
AIDA	0.61087	0.01556
HOOK3	0.60177	0.01762
AKR1C2	0.60173	0.01763
AKR1C1	0.59989	0.01808
EEF1D	-0.5993	0.01822
TNFRSF1A	0.59028	0.02053
RPL35	-0.58725	0.02135
FOLR1	0.58635	0.0216
GLS	0.58601	0.02169
KRT7	0.58042	0.0233
KRTCAP2	0.58039	0.02331
MAPKAPK2	0.57974	0.0235
RPS25	-0.57923	0.02365
LAMP1	0.57904	0.02371
RAB5B	0.57686	0.02436
CERS2	0.57006	0.02651
JTB	0.56711	0.02748
TKT	0.56656	0.02766
RPN2	0.56539	0.02806
KTN1	0.56467	0.0283
VAPA	0.56004	0.02992
RPS13	-0.55991	0.02996
ATP1B1	0.55889	0.03033

**RNF145**	0.55877	0.03037
**FASN**	0.55813	0.0306
**LMNA**	0.557	0.03101
**RPS15**	-0.55555	0.03155
**TOB1**	0.55405	0.03211
**ADIPOR1**	0.55274	0.0326
**MANF**	0.5499	0.03369
**ASAH1**	0.54976	0.03375
**MCL1**	0.54513	0.03559
**XRCC6**	0.54491	0.03568
**FOS**	0.54489	0.03569
**NENF**	0.54241	0.03671
**ALCAM**	0.54225	0.03677
**CXCL16**	0.54061	0.03746
**CBR1**	0.5361	0.0394
**FOSB**	0.53549	0.03967
**RASD1**	0.53397	0.04034
**MSLN**	0.5338	0.04041
**PTMA**	-0.53196	0.04124
**RPS5**	-0.53074	0.0418
**ADAM15**	0.52911	0.04255
**SCNN1A**	0.52848	0.04284
**TSR3**	-0.52515	0.04441
**TM9SF2**	0.52512	0.04443
**SUMF2**	0.52371	0.04511
**ALDH3A2**	0.52156	0.04615
**PGD**	0.51902	0.04742
**ERBB3**	0.51842	0.04772
**PBXIP1**	0.51715	0.04836
**PTP4A2**	0.51532	0.0493
**ANXA5**	0.5152	0.04936
