# Supplementary material for: The Effect of Exposure to Neighborhood Violence on Glucocorticoid Receptor Signaling in Lung Tumors
Source: Cancer Res Commun. 2024 Jul 3;4(7):1643–54. doi: 10.1158/2767-9764.CRC-24-0032 (PMC11221527; doi:10.1158/2767-9764.CRC-24-0032)
Supplement: Supplementary Table S2 — Total binding site number in tumor and normal tissue samples. [file crc-24-0032_supplementary_table_s2_suppst2.pdf]

Supplementary Table 2. Total binding site number in tumor and normal tissue samples.

Sample Number	Number of Binding Sites: Tumor binding sites with input binding sites filtered out	Number of Binding Sites: Normal Tissue binding sites with input binding sites filtered out	Number of Binding Sites: Tumor binding sites with normal tissue binding sites filtered out	Number of Binding Sites: Normal Tissue binding sites with tumor binding sites filtered out
**1**	5416	6036	4387	6520
**2**	2798	19694	109	20304
**3**	8576	2017	7829	2644
**4**	1915	1725	1781	191
**6**	15347	17167	14109	10
**7**	1411	1313	1368	19641
**8**	1094	2773	1232	1242
**9**	2220	7874	124	102
**11**	1546	5861	1589	8007
**12**	76763	7437	76763	5
**13**	907	4601	965	7030
**14**	3884	4484	3130	3249
**15**	4035	949	6405	998
**16**	2376	5592	1943	6941
