# Supplementary material for: The Effect of Exposure to Neighborhood Violence on Glucocorticoid Receptor Signaling in Lung Tumors
Source: Cancer Res Commun. 2024 Jul 3;4(7):1643–54. doi: 10.1158/2767-9764.CRC-24-0032 (PMC11221527; doi:10.1158/2767-9764.CRC-24-0032)
Supplement: Supplementary Table S3 — Pathway analysis results of cluster 6 genes from Figure 4A. [file crc-24-0032_supplementary_table_s3_suppst3.pdf]

Supplementary Table 3. Pathway analysis results of cluster 6 genes from Figure 4A.

Category	Term	P-Value	Fold Enrichment	Bonferroni	Benjamini	FDR
WIKIPATHWAYS	WP4659~Gastrin signaling pathway	0.02	11.93959	0.954296	1	1
WIKIPATHWAYS	WP4928~MAPK pathway in congenital thyroid cancer	0.03	57.21053	0.987474	1	1
WIKIPATHWAYS	WP481~Insulin signaling	0.04	8.581579	0.996738	1	1
WIKIPATHWAYS	WP437~EGF/EGFR signaling pathway	0.04	8.475634	0.997145	1	1
WIKIPATHWAYS	WP3888~VEGFA-VEGFR2 signaling	0.06	4.237817	0.99957	1	1
WIKIPATHWAYS	WP3972~PDGFR-beta pathway	0.06	31.56443	0.999645	1	1
BIOCARTA	h_il3Pathway:IL 3 signaling pathway	0.06	27.05	0.981167	0.626684	0.626684
WIKIPATHWAYS	WP5269~Genetic causes of porto-sinusoidal vascular disease	0.07	24.73969	0.999961	1	1
BIOCARTA	h_epoPathway:EPO Signaling Pathway	0.08	21.35526	0.993511	0.626684	0.626684
WIKIPATHWAYS	WP2526~PDGF pathway	0.08	22.88421	0.999983	1	1
WIKIPATHWAYS	WP127~IL-5 signaling pathway	0.08	22.88421	0.999983	1	1
BIOCARTA	h_ngfPathway:Nerve growth factor pathway (NGF)	0.08	20.2875	0.99503	0.626684	0.626684
WIKIPATHWAYS	WP49~IL-2 signaling pathway	0.08	21.79449	0.99999	1	1
BIOCARTA	h_il6Pathway:IL 6 signaling pathway	0.09	19.32143	0.996195	0.626684	0.626684
BIOCARTA	h_insulinPathway:Insulin Signaling Pathway	0.09	19.32143	0.996195	0.626684	0.626684
BIOCARTA	h_igf1Pathway:IGF-1 Signaling Pathway	0.09	19.32143	0.996195	0.626684	0.626684
BIOCARTA	h_il2Pathway:IL 2 signaling pathway	0.09	18.44318	0.997087	0.626684	0.626684
WIKIPATHWAYS	WP286~IL-3 signaling pathway	0.10	18.30737	0.999999	1	1
BIOCARTA	h_tpoPathway:TPO Signaling Pathway	0.10	16.90625	0.998293	0.626684	0.626684

BIOCARTA	h_ecmPathway:Erk and PI-3 Kinase Are Necessary for Collagen Binding in Corneal Epithelia	0.10	16.90625	0.998293	0.626684	0.626684

Genes were annotated using GREAT analysis and pathway analysis was performed in DAVID using Biocarta, Kegg, and Wikipathways analysis.
