# Supplementary material for: The Effect of Exposure to Neighborhood Violence on Glucocorticoid Receptor Signaling in Lung Tumors
Source: Cancer Res Commun. 2024 Jul 3;4(7):1643–54. doi: 10.1158/2767-9764.CRC-24-0032 (PMC11221527; doi:10.1158/2767-9764.CRC-24-0032)
Supplement: Supplementary Table S4 — Pathway analysis results of cluster 7 genes from Figure 4A. [file crc-24-0032_supplementary_table_s4_suppst4.pdf]

Supplementary Table 4. Pathway analysis results of cluster 7 genes from Figure 4A.

Category	Term	P-Value	Fold Enrichment	Bonferroni	Benjamini	FDR
**WIKIPATHWAYS**	WP4493~Cells and molecules involved in local acute inflammatory response	0.09	21.76721	1	1	1
WIKIPATHWAYS	WP3670~Interactions between LOXL4 and oxidative stress pathway	0.09	20.55792	1	1	1
WIKIPATHWAYS	WP5300~TROP2 regulatory signaling	0.03	11.32783	0.994564	1	1
WIKIPATHWAYS	WP560~TGF-beta receptor signaling	0.03	10.09207	0.998461	1	1
WIKIPATHWAYS	WP558~Complement and coagulation cascades	0.04	9.570066	0.999216	1	1
WIKIPATHWAYS	WP4816~TGF-beta receptor signaling in skeletal dysplasias	0.04	9.407862	0.999378	1	1
KEGG_PATHWAY	hsa04610:Complement and coagulation cascades	0.06	7.305162	0.99989	1	1
KEGG_PATHWAY	hsa04512:ECM-receptor interaction	0.06	7.05892	0.999939	1	1
WIKIPATHWAYS	WP5322~CKAP4 signaling pathway map	0.02	6.380044	0.987511	1	1
KEGG_PATHWAY	hsa04933:AGE-RAGE signaling pathway in diabetic complications	0.08	6.282439	0.999994	1	1
KEGG_PATHWAY	hsa04659:Th17 cell differentiation	0.09	5.817073	0.999999	1	1
WIKIPATHWAYS	WP3888~VEGFA-VEGFR2 signaling	0.00	4.2829	0.065381	0.067965	0.067965
KEGG_PATHWAY	hsa05415:Diabetic cardiomyopathy	0.07	4.126397	0.999965	1	1
KEGG_PATHWAY	hsa05020:Prion disease	0.04	3.849534	0.995839	1	1
KEGG_PATHWAY	hsa04714:Thermogenesis	0.09	3.610597	0.999999	1	1
KEGG_PATHWAY	hsa05165:Human papillomavirus infection	0.07	3.163363	0.999959	1	1
KEGG_PATHWAY	hsa04151:PI3K-Akt signaling pathway	0.08	2.916638	0.999997	1	1

KEGG_PATHWAY	hsa05014:Amyotrophic lateral sclerosis	0.09	2.876575	0.999999	1	1
KEGG_PATHWAY	hsa05200:Pathways in cancer	0.03	2.760645	0.994284	1	1

Genes were annotated using GREAT analysis and pathway analysis was performed in DAVID using Biocarta, Kegg, and Wikipathways analysis.
