# Supplementary material for: The Effect of Exposure to Neighborhood Violence on Glucocorticoid Receptor Signaling in Lung Tumors
Source: Cancer Res Commun. 2024 Jul 3;4(7):1643–54. doi: 10.1158/2767-9764.CRC-24-0032 (PMC11221527; doi:10.1158/2767-9764.CRC-24-0032)
Supplement: Supplementary Table S5 — Pathway analysis results of cluster 7 genes from Supplementary Figure 4A. [file crc-24-0032_supplementary_table_s5_suppst5.pdf]

Supplementary Table 5. Pathway analysis results of cluster 7 genes from Supplementary Figure 4A.

Category	Term	P-Value	Fold Enrichment	Bonferroni	Benjamini	FDR
**WIKIPATHWAYS**	WP2431~Spinal cord injury	0.004	5.600086	0.652148	0.543767	0.543767
WIKIPATHWAYS	WP3888~VEGFA-VEGFR2 signaling	0.004	2.875661	0.662314	0.543767	0.543767
WIKIPATHWAYS	WP3969~H19 action Rb-E2F1 signaling and CDK-Beta-catenin activity	0.007	22.58701	0.854306	0.557337	0.557337
KEGG_PATHWAY	hsa04670:Leukocyte transendothelial migration	0.011	5.656126	0.868961	1	1
WIKIPATHWAYS	WP5144~NRP1-triggered signaling pathways in pancreatic cancer	0.012	8.36556	0.951956	0.557337	0.557337
WIKIPATHWAYS	WP2064~Neural crest differentiation	0.012	5.590845	0.952605	0.557337	0.557337
WIKIPATHWAYS	WP3646~Hepatitis C and hepatocellular carcinoma	0.013	8.06679	0.96501	0.557337	0.557337
KEGG_PATHWAY	hsa05220:Chronic myeloid leukemia	0.020	6.84689	0.973607	1	1
WIKIPATHWAYS	WP4905~1q21.1 copy number variation syndrome	0.026	11.68294	0.999042	0.983651	0.983651
WIKIPATHWAYS	WP5094~Orexin receptor pathway	0.031	3.371196	0.999724	1	1
KEGG_PATHWAY	hsa04514:Cell adhesion molecules	0.031	4.116801	0.997094	1	1
KEGG_PATHWAY	hsa04520:Adherens junction	0.033	5.595308	0.997916	1	1
KEGG_PATHWAY	hsa04141:Protein processing in endoplasmic reticulum	0.039	3.826203	0.999373	1	1
WIKIPATHWAYS	WP4844~Influence of laminopathies on Wnt signaling	0.041	9.156897	0.999983	1	1
WIKIPATHWAYS	WP3614~Photodynamic therapy-induced HIF-1 survival signaling	0.043	8.915926	0.99999	1	1
WIKIPATHWAYS	WP138~Androgen receptor signaling pathway	0.045	4.964179	0.999994	1	1
KEGG_PATHWAY	hsa04659:Th17 cell differentiation	0.048	4.818182	0.999882	1	1
WIKIPATHWAYS	WP399~Wnt signaling pathway and pluripotency	0.060	4.428826	1	1	1

BIOCARTA	H_gsk3Pathway:Inactivation of Gsk3 by AKT causes accumulation of b-catenin in Alveolar Macrophages	0.063	6.935897	0.980127	1	1
WIKIPATHWAYS	WP1591~Heart development	0.063	7.208621	1	1	1
KEGG_PATHWAY	hsa04919:Thyroid hormone signaling pathway	0.064	4.300526	0.999994	1	1
WIKIPATHWAYS	WP5300~TROP2 regulatory signaling	0.068	6.914392	1	1	1
KEGG_PATHWAY	hsa05205:Proteoglycans in cancer	0.069	3.172949	0.999998	1	1
WIKIPATHWAYS	WP363~Wnt signaling pathway	0.073	6.643239	1	1	1
WIKIPATHWAYS	WP2272~Pathogenic Escherichia coli infection	0.083	6.160094	1	1	1
KEGG_PATHWAY	hsa05166:Human T-cell leukemia virus 1 infection	0.087	2.929975	1	1	1
WIKIPATHWAYS	WP4816~TGF-beta receptor signaling in skeletal dysplasias	0.094	5.742461	1	1	1
WIKIPATHWAYS	WP411~mRNA processing	0.098	3.58524	1	1	1

Genes were annotated using GREAT analysis and pathway analysis was performed in DAVID using Biocarta, Kegg, and Wikipathways analysis.
