# Supplementary material for: The Effect of Exposure to Neighborhood Violence on Glucocorticoid Receptor Signaling in Lung Tumors
Source: Cancer Res Commun. 2024 Jul 3;4(7):1643–54. doi: 10.1158/2767-9764.CRC-24-0032 (PMC11221527; doi:10.1158/2767-9764.CRC-24-0032)
Supplement: Supplementary Table S6 — Pathway analysis results of cluster 6 genes from Supplementary Figure 4B. [file crc-24-0032_supplementary_table_s6_suppst6.pdf]

Supplementary Table 6. Pathway analysis results of cluster 6 genes from Supplementary Figure 4B.

**Category**	**Term**	**P-Value**	**Fold Enrichment**	**Bonferroni**	**Benjamini**	**FDR**
**KEGG_PATHWAY**	hsa04310:Wnt signaling pathway	5.64E-05	2.552319	0.017999	0.018162	0.018162
WIKIPATHWAYS	WP2118~Arrhythmogenic right ventricular cardiomyopathy	1.92E-04	3.395927	0.09987	0.050998	0.05072
KEGG_PATHWAY	hsa05412:Arrhythmogenic right ventricular cardiomyopathy	2.13E-04	3.36442	0.066153	0.034218	0.034218
WIKIPATHWAYS	WP2857~Mesodermal commitment pathway	2.73E-04	2.480791	0.139109	0.050998	0.05072
WIKIPATHWAYS	WP5052~Nephrogenesis	2.78E-04	7.074849	0.141644	0.050998	0.05072
BIOCARTA	h_gabaPathway:Gamma-aminobutyric Acid Receptor Life Cycle	3.65E-04	8.273577	0.060119	0.061626	0.061626
WIKIPATHWAYS	WP5066~FOXA2 pathway	7.06E-04	6.064156	0.321508	0.097113	0.096583
KEGG_PATHWAY	hsa04015:Rap1 signaling pathway	9.15E-04	2.114778	0.255177	0.098158	0.098158
BIOCARTA	h_FlumazenilPathway:Cardiac Protection Against ROS	0.002	8.426791	0.248548	0.141914	0.141914
WIKIPATHWAYS	WP5087~Pleural mesothelioma	0.002	1.653861	0.650704	0.175745	0.174786
WIKIPATHWAYS	WP2858~Ectoderm differentiation	0.002	2.306088	0.651306	0.175745	0.174786
KEGG_PATHWAY	hsa04020:Calcium signaling pathway	0.002	1.901629	0.536349	0.191927	0.191927
KEGG_PATHWAY	hsa04360:Axon guidance	0.004	2.033441	0.746653	0.274014	0.274014

WIKIPATHWAYS	WP4787~Osteoblast differentiation and related diseases	0.005	2.293168	0.950336	0.428534	0.426196
WIKIPATHWAYS	WP4540~Hippo signaling regulation pathways	0.007	2.413287	0.980165	0.442842	0.440426
WIKIPATHWAYS	WP4148~Splicing factor NOVA regulated synaptic proteins	0.007	3.465232	0.981552	0.442842	0.440426
WIKIPATHWAYS	WP4823~Genes controlling nephrogenesis	0.009	3.307722	0.994303	0.461988	0.459468
WIKIPATHWAYS	WP5117~Cohesin complex - Cornelia de Lange syndrome	0.010	3.745508	0.994876	0.461988	0.459468
WIKIPATHWAYS	WP428~Wnt signaling	0.010	2.214735	0.996158	0.461988	0.459468
BIOCARTA	h_myosinPathway:PKC-catalyzed phosphorylation of inhibitory phosphoprotein of myosin phosphatase	0.010	5.417223	0.829051	0.582303	0.582303
WIKIPATHWAYS	WP2853~Endoderm differentiation	0.012	2.021385	0.998907	0.522203	0.519354
KEGG_PATHWAY	hsa00062:Fatty acid elongation	0.013	4.112069	0.987201	0.643778	0.643778
WIKIPATHWAYS	WP5231~Hippocampal synaptogenesis and neurogenesis	0.014	4.042771	0.999652	0.56564	0.562554
KEGG_PATHWAY	hsa00562:Inositol phosphate metabolism	0.016	2.534837	0.994577	0.643778	0.643778
KEGG_PATHWAY	hsa04371:Apelin signaling pathway	0.018	1.996868	0.996643	0.643778	0.643778
KEGG_PATHWAY	hsa04022:cGMP-PKG signaling pathway	0.018	1.883672	0.997111	0.643778	0.643778
KEGG_PATHWAY	hsa04810:Regulation of actin cytoskeleton	0.022	1.696902	0.999322	0.708431	0.708431
WIKIPATHWAYS	WP5402~10q22q23 copy number variation	0.023	2.558316	0.999997	0.841705	0.837113

KEGG_PATHWAY	hsa05032:Morphine addiction	0.024	2.236785	0.999625	0.708431	0.708431
WIKIPATHWAYS	WP4159~GABA receptor signaling	0.025	3.521123	0.999999	0.868699	0.86396
WIKIPATHWAYS	WP35~G protein signaling pathways	0.027	2.19909	1	0.868699	0.86396
WIKIPATHWAYS	WP560~TGF-beta receptor signaling	0.030	2.646177	1	0.906865	0.901918
WIKIPATHWAYS	WP4541~Hippo-Merlin signaling dysregulation	0.033	1.954563	1	0.9619	0.956654
WIKIPATHWAYS	WP5321~Prostaglandin and leukotriene metabolism in senescence	0.036	3.210436	1	0.998599	0.993152
WIKIPATHWAYS	WP4816~TGF-beta receptor signaling in skeletal dysplasias	0.041	2.466775	1	1	0.996357
WIKIPATHWAYS	WP5053~Development of ureteric collection system	0.045	2.425662	1	1	0.996357
KEGG_PATHWAY	hsa04916:Melanogenesis	0.045	2.015321	1	1	1
KEGG_PATHWAY	hsa04330:Notch signaling pathway	0.048	2.387653	1	1	1
WIKIPATHWAYS	WP2064~Neural crest differentiation	0.050	1.981358	1	1	0.996357
WIKIPATHWAYS	WP399~Wnt signaling pathway and pluripotency	0.053	1.961933	1	1	0.996357
WIKIPATHWAYS	WP4962~Airway smooth muscle cell contraction	0.054	4.548117	1	1	0.996357
WIKIPATHWAYS	WP4829~mBDNF and proBDNF regulation of GABA neurotransmission	0.055	2.872495	1	1	0.996357
WIKIPATHWAYS	WP3931~Embryonic stem cell pluripotency pathways	0.056	1.865894	1	1	0.996357
WIKIPATHWAYS	WP4336~ncRNAs involved in Wnt signaling in hepatocellular carcinoma	0.058	2.021385	1	1	0.996357

KEGG_PATHWAY	hsa05226:Gastric cancer	0.059	1.73866	1	1	1
WIKIPATHWAYS	WP363~Wnt signaling pathway	0.059	2.497005	1	1	0.996357
KEGG_PATHWAY	hsa04928:Parathyroid hormone synthesis, secretion and action	0.059	1.920259	1	1	1
BIOCARTA	h_rhoPathway:Rho cell motility signaling pathway	0.059	2.757859	0.999969	1	1
KEGG_PATHWAY	hsa04024:cAMP signaling pathway	0.060	1.562586	1	1	1
KEGG_PATHWAY	hsa05033:Nicotine addiction	0.062	2.775647	1	1	1
WIKIPATHWAYS	WP3678~Amplification and expansion of oncogenic pathways as metastatic traits	0.063	4.280581	1	1	0.996357
KEGG_PATHWAY	hsa05200:Pathways in cancer	0.064	1.324226	1	1	1
WIKIPATHWAYS	WP3651~Pathways affected in adenoid cystic carcinoma	0.064	2.239073	1	1	0.996357
KEGG_PATHWAY	hsa05414:Dilated cardiomyopathy	0.073	1.927532	1	1	1
KEGG_PATHWAY	hsa04070:Phosphatidylinositol signaling system	0.077	1.907661	1	1	1
WIKIPATHWAYS	WP2855~Dopaminergic neurogenesis	0.079	3.032078	1	1	0.996357
KEGG_PATHWAY	hsa04120:Ubiquitin mediated proteolysis	0.081	1.694057	1	1	1
WIKIPATHWAYS	WP4262~Breast cancer pathway	0.083	1.643191	1	1	0.996357
WIKIPATHWAYS	WP4249~Hedgehog signaling pathway	0.085	2.538484	1	1	0.996357

WIKIPATHWAYS	WP5122~Prostaglandin and leukotriene metabolism in senescence	0.087	2.934269	1	1	0.996357
WIKIPATHWAYS	WP4258~lncRNA in canonical Wnt signaling and colorectal cancer	0.088	1.856374	1	1	0.996357
WIKIPATHWAYS	WP4879~Overlap between signal transduction pathways contributing to LMNA laminopathies	0.091	2.234163	1	1	0.996357
KEGG_PATHWAY	hsa04742:Taste transduction	0.091	1.936498	1	1	1
WIKIPATHWAYS	WP2363~Gastric cancer network 2	0.096	2.842573	1	1	0.996357
WIKIPATHWAYS	WP2839~Hair follicle development: organogenesis - part 2 of 3	0.096	2.842573	1	1	0.996357
KEGG_PATHWAY	hsa05224:Breast cancer	0.099	1.636436	1	1	1
KEGG_PATHWAY	hsa01210:2-Oxocarboxylic acid metabolism	0.100	2.803683	1	1	1

Genes were annotated using GREAT analysis and pathway analysis was performed in DAVID using Biocarta, Kegg, and Wikipathways analysis.
