# Supplementary material for: The Effect of Exposure to Neighborhood Violence on Glucocorticoid Receptor Signaling in Lung Tumors
Source: Cancer Res Commun. 2024 Jul 3;4(7):1643–54. doi: 10.1158/2767-9764.CRC-24-0032 (PMC11221527; doi:10.1158/2767-9764.CRC-24-0032)
Supplement: Supplementary Table S7 — Pathway analysis results of cluster 6 genes from Supplementary Figure 4C. [file crc-24-0032_supplementary_table_s7_suppst7.pdf]

Supplementary Table 7. Pathway analysis results of cluster 6 genes from Supplementary Figure 4C.

Category	Term	P-Value	Fold Enrichment	Bonferroni	Benjamini	FDR
**KEGG_PATHWAY**	hsa04080:Neuroactive ligand-receptor interaction	2.98E-04	1.8969	0.087476	0.091528	0.091528
WIKIPATHWAYS	WP2857~Mesodermal commitment pathway	4.30E-04	2.395592	0.214784	0.104745	0.104745
WIKIPATHWAYS	WP2853~Endoderm differentiation	4.87E-04	2.439955	0.239328	0.104745	0.104745
WIKIPATHWAYS	WP5352~10q11.21q11.23 copy number variation syndrome	5.58E-04	3.455936	0.269308	0.104745	0.104745
KEGG_PATHWAY	hsa04360:Axon guidance	0.001	2.231289	0.285189	0.167776	0.167776
WIKIPATHWAYS	WP5053~Development of ureteric collection system	0.002	3.220741	0.646157	0.241776	0.241776
WIKIPATHWAYS	WP58~Monoamine GPCRs	0.002	4.258831	0.701215	0.241776	0.241776
KEGG_PATHWAY	hsa04024:cAMP signaling pathway	0.003	1.976757	0.585613	0.29323	0.29323
WIKIPATHWAYS	WP4830~GDNF/RET signaling axis	0.008	4.582872	0.990832	0.780142	0.780142
KEGG_PATHWAY	hsa04020:Calcium signaling pathway	0.011	1.757985	0.968147	0.782412	0.782412
WIKIPATHWAYS	WP536~Calcium regulation in cardiac cells	0.012	1.964806	0.999082	0.994619	0.994619
KEGG_PATHWAY	hsa05412:Arrhythmogenic right ventricular cardiomyopathy	0.017	2.511408	0.994994	0.782412	0.782412
WIKIPATHWAYS	WP5236~Markers of kidney cell lineage	0.018	2.679815	0.999955	1	1
KEGG_PATHWAY	hsa00510:N-Glycan biosynthesis	0.018	2.918919	0.996588	0.782412	0.782412

WIKIPATHWAYS	WP2637~Interleukin-1 (IL-1) structural pathway	0.020	2.868192	0.999987	1	1
KEGG_PATHWAY	hsa04970:Salivary secretion	0.021	2.287271	0.998584	0.782412	0.782412
KEGG_PATHWAY	hsa04350:TGF-beta signaling pathway	0.023	2.148649	0.999246	0.782412	0.782412
KEGG_PATHWAY	hsa04924:Renin secretion	0.025	2.522327	0.999574	0.782412	0.782412
KEGG_PATHWAY	hsa04022:cGMP-PKG signaling pathway	0.025	1.852727	0.999639	0.782412	0.782412
WIKIPATHWAYS	WP2406~Cardiac progenitor differentiation	0.029	2.651725	1	1	1
WIKIPATHWAYS	WP399~Wnt signaling pathway and pluripotency	0.030	2.066786	1	1	1
KEGG_PATHWAY	hsa04310:Wnt signaling pathway	0.035	1.778192	0.999982	0.974963	0.974963
WIKIPATHWAYS	WP481~Insulin signaling	0.038	1.756768	1	1	1
KEGG_PATHWAY	hsa04974:Protein digestion and absorption	0.039	2.065206	0.999996	0.974963	0.974963
WIKIPATHWAYS	WP5153~N-glycan biosynthesis	0.041	2.465639	1	1	1
WIKIPATHWAYS	WP3944~Serotonin and anxiety-related events	0.042	5.019336	1	1	1
WIKIPATHWAYS	WP4321~Thermogenesis	0.042	1.951964	1	1	1
KEGG_PATHWAY	hsa04950:Maturity onset diabetes of the young	0.042	3.718815	0.999998	0.974963	0.974963
WIKIPATHWAYS	WP2880~Glucocorticoid receptor pathway	0.044	2.258701	1	1	1
KEGG_PATHWAY	hsa05032:Morphine addiction	0.044	2.125037	0.999999	0.974963	0.974963
WIKIPATHWAYS	WP4258~lncRNA in canonical Wnt signaling and colorectal cancer	0.051	1.971882	1	1	1

WIKIPATHWAYS	WP410~Exercise-induced circadian regulation	0.053	2.561953	1	1	1
KEGG_PATHWAY	hsa05414:Dilated cardiomyopathy	0.059	2.014358	1	1	1
KEGG_PATHWAY	hsa04010:MAPK signaling pathway	0.061	1.477642	1	1	1
WIKIPATHWAYS	WP2118~Arrhythmogenic right ventricular cardiomyopathy	0.062	2.108121	1	1	1
WIKIPATHWAYS	WP3591~Sleep regulation	0.062	2.773844	1	1	1
WIKIPATHWAYS	WP4808~Endochondral ossification with skeletal dysplasias	0.065	2.230816	1	1	1
WIKIPATHWAYS	WP474~Endochondral ossification	0.065	2.230816	1	1	1
KEGG_PATHWAY	hsa00230:Purine metabolism	0.066	1.812922	1	1	1
WIKIPATHWAYS	WP363~Wnt signaling pathway	0.068	2.41125	1	1	1
WIKIPATHWAYS	WP3947~Serotonin and anxiety	0.068	4.133571	1	1	1
KEGG_PATHWAY	hsa04724:Glutamatergic synapse	0.073	1.849706	1	1	1
KEGG_PATHWAY	hsa04911:Insulin secretion	0.075	2.023727	1	1	1
KEGG_PATHWAY	hsa04015:Rap1 signaling pathway	0.076	1.565444	1	1	1
WIKIPATHWAYS	WP3967~miR-509-3p alteration of YAP1/ECM axis	0.079	3.903928	1	1	1
WIKIPATHWAYS	WP117~GPCRs, other	0.081	1.888998	1	1	1
WIKIPATHWAYS	WP5205~Clock-controlled autophagy in bone metabolism	0.083	1.976364	1	1	1
WIKIPATHWAYS	WP2855~Dopaminergic neurogenesis	0.088	2.927946	1	1	1
BIOCARTA	h_wntPathway:WNT Signaling Pathway	0.089	2.863444	1	1	1
WIKIPATHWAYS	WP247~Small ligand GPCRs	0.090	3.698458	1	1	1
WIKIPATHWAYS	WP2795~Cardiac hypertrophic response	0.090	2.235886	1	1	1
BIOCARTA	h_alkPathway:ALK in cardiac myocytes	0.094	2.41458	1	1	1
KEGG_PATHWAY	hsa04928:Parathyroid hormone synthesis, secretion and action	0.096	1.824324	1	1	1

WIKIPATHWAYS	WP4304~Oligodendrocyte specification and differentiation, leading to myelin components for CNS	0.096	2.833496	1	1	1
WIKIPATHWAYS	WP4222~Phosphodiesterases in neuronal function	0.097	2.19596	1	1	1

Genes were annotated using GREAT analysis and pathway analysis was performed in DAVID using Biocarta, Kegg, and Wikipathways analysis.
